# Causes and Outcomes of Admission and Investigation of Tuberculosis in Adults with Advanced HIV in South African Hospitals: Data from the TB Fast Track Trial

**DOI:** 10.4269/ajtmh.21-0133

**Published:** 2021-10-18

**Authors:** Peter G. Beckwith, Mpho Tlali, Salome Charalambous, Gavin J. Churchyard, Katherine L. Fielding, Christopher J. Hoffmann, Suzanne Johnson, Natalie Wood, Alison D. Grant, Aaron S. Karat

**Affiliations:** ^1^ TB Centre, London School of Hygiene & Tropical Medicine, London, United Kingdom;; ^2^ Department of Medicine, University of Cape Town, Cape Town, South Africa;; ^3^ The Aurum Institute, Johannesburg, South Africa;; ^4^ School of Public Health, University of the Witwatersrand, Johannesburg, South Africa;; ^5^ Advancing Care and Treatment for TB and HIV, South African Medical Research Council. Johannesburg, South Africa;; ^6^ Johns Hopkins University School of Medicine, Baltimore, Maryland;; ^7^ Foundation for Professional Development, Pretoria, South Africa;; ^8^ North Bristol NHS Trust, Bristol, United Kingdom;; ^9^ Africa Health Research Institute, School of Laboratory Medicine & Medical Sciences, College of Health Sciences, University of KwaZulu-Natal, Durban, South Africa

## Abstract

Tuberculosis (TB) remains the leading cause of hospitalization and in-hospital mortality in HIV-positive adults. Using data from hospital and clinic files, research databases, and autopsy, we describe causes and outcomes of admissions, and assess investigations for TB among adults with advanced HIV who were hospitalized after enrollment into the TB Fast Track trial in South Africa (2013–2015). A total of 251 adults [median CD4 count, 37.5 cells/μL; interquartile range, 14–68 cells/µL; 152 (60.6%) on antiretroviral therapy] experienced 304 admissions. Ninety-five of 251 of the first admissions (37.8%) were TB related; the next most common causes were AIDS-related illnesses (41 of 251, 16.3%) and surgical causes (21 of 251, 8.4%). Of those admitted with previously undiagnosed TB, 60% had CD4 counts less than 50 cells/µL. Overall, 137 of 251 individuals died as inpatients or within 90 days of their first discharge. Case fatality rates were particularly high for those admitted with TB (66%) and bacterial infections (80%). In 144 admissions for whom anti-TB treatment had not been started before admission, a sputum-based TB investigation was recorded in only 12 of 57 admissions (21.1%) in whom one or more TB symptom was recorded (24 of 57 started on treatment), and 6 of 87 admissions (6.9%) in whom no TB symptoms were recorded (14 of 87 started on treatment). Hospitalized adults with advanced HIV are at high risk of death. TB was a common cause of hospitalization but was under-investigated, even in those with symptoms. In addition to early identification of TB and other AIDS-related illnesses during hospitalization of adults with advanced HIV, improved pre-hospital management strategies are needed to interrupt disease progression and reduce poor outcomes in this already vulnerable population.

## INTRODUCTION

Despite increased availability of antiretroviral therapy (ART), adults with advanced HIV (CD4 count, < 200 cells/µL, or WHO clinical stage 3 or 4 at presentation)^1^ are at high risk of poor outcomes.^2^ Mortality in hospitalized adults with advanced HIV in sub-Saharan Africa ranges from 7% to 44%,^3^^–^^7^ and 25% to 40% of adults initiated on ART in low- and middle-income countries have advanced HIV at the time of initiation.^8^ It is therefore critical to understand the reasons for continued poor outcomes in this vulnerable group, but this is not straightforward, as routine data on morbidity and mortality in individuals with advanced HIV are not widely available in most countries most affected by the HIV epidemic.

The WHO recommends timely ART initiation and early management of opportunistic infections as part of an integrated package of care for individuals with advanced HIV.^1^ An important diagnostic element of this package is to test sputum for tuberculosis (TB) using the Xpert MTB/RIF assay for all symptomatic adults,^9^ and to test urine using lateral-flow lipoarabinomannan (LF-LAM) in adults with CD4 counts less than 100 cells/µL. This is necessary because TB remains the leading cause of hospitalization and in-hospital mortality in HIV-positive adults worldwide,^10^ and autopsy studies show that around 50% of TB goes undiagnosed in hospitalized HIV-positive individuals.^11^^,^^12^

Screening HIV-positive adults for TB using the WHO four clinical symptoms (current cough, fever, night sweats, or weight loss) is 78.9% (95% CI, 58–91%) sensitive.^13^ However, the negative predictive value is lower in settings with a greater TB prevalence.^13^ In two studies in South Africa, Lawn et al.^14^^,^^15^ demonstrated that symptoms were not predictive for TB in adults admitted to the hospital, suggesting a need for routine TB testing for all HIV-positive adults admitted to medical wards in high TB-burden settings, regardless of presenting complaints.^14^^,^^16^ Although LF-LAM has been recommended for use,^17^ it has not yet been implemented routinely in hospitals in South Africa. Therefore, it remains relevant to develop our understanding more fully regarding the utility of sputum investigations in adults with advanced HIV.

We aimed to estimate the causes and outcomes of admission, and assess the investigation and treatment of TB during admission, of the ambulatory adults with advanced HIV who were recruited to the TB Fast Track trial at 24 primary health-care (PHC) clinics in South Africa and were subsequently admitted to the hospital between 2013 and 2015.^18^

## METHODS

### Parent trial.

Our analysis used data from adults with advanced HIV who were admitted to the hospital up to 1 year after enrollment to TB Fast Track—a pragmatic, cluster-randomized trial of empirical TB treatment of patients attending PHC clinics in South Africa. Detailed trial methods have been reported previously.^18^^,^^19^ In brief, HIV-positive adults were recruited to TB Fast Track at 24 PHC clinics; 12 clinics were allocated randomly to each of the intervention and control arms. Participants were 18 years or older, with CD4 counts of ≤150 cells/µL, and had not received TB treatment or ART in the previous 3 or 6 months, respectively. In the intervention arm, each participant was assessed for their probability of active TB using the study algorithm and was initiated on empirical TB treatment as soon as possible if considered “high probability.” Participants in the control arm were managed by clinic staff in line with their usual practices. There were seven public referral hospitals surrounding the 24 PHCs. Inpatient management was not part of the intervention, and the study team did not capture admissions in real time or influence the management of hospitalized participants.

### Context.

The Xpert MTB/RIF assay was rolled out in South Africa from 2011 to 2013 as the first-line TB diagnostic test. At the time of the study (and still today), national guidelines recommended an Xpert MTB/RIF on sputum for all individuals with symptoms suggestive of TB.^20^ In HIV-positive adults, a chest radiograph, second sputum for culture, and course of antibiotics are suggested if the initial Xpert MTB/RIF is negative.^21^ Urine LF-LAM had not been recommended for hospitalized adults with advanced HIV at the time of the study and has not yet been implemented in hospitals in South Africa. Patients were referred from PHC facilities to district or tertiary level hospitals when inpatient management was needed.

### Notification of hospitalization and collection of data from hospitals and laboratories.

All participants were asked by study staff at follow-up visits if they had attended a hospital at any point after enrollment. Study staff also looked for evidence of hospitalizations when abstracting data from clinic files. For each participant with a recorded hospitalization and for all participants who were recorded as having died after enrollment, the study team searched the admission records manually of the surrounding public hospitals and reviewed clinical notes when they could be located. The National Health Laboratory Service (NHLS) database was also searched and the results of investigations recorded. Data were abstracted by a member of the study team (doctor or professional nurse) using standardized paper forms. Ascertainment of death was done as part of the main trial and has been described previously in detail.^18^

### Data management.

Records of participants’ hospitalization were maintained in a password-protected Microsoft Excel workbook (Version 2108, Microsoft Corporation, Redmond, WA). Anonymized data from hospital files and the NHLS database were maintained in EpiData databases (EpiData Association, Odense, Denmark). Relevant data from the parent trial and an autopsy sub-study were also used to inform the analysis.^22^ The data are available at https://datacompass.lshtm.ac.uk/.

### Analysis.

#### Inclusion and exclusion criteria.

An admission was defined as spending at least one night in a hospital or death in a hospital on the day of attendance. Admissions from both trial arms that occurred from enrollment up to 1 year after enrollment were included. Characteristics of admissions were only assessed for individuals’ first admission. Data from individuals not on TB treatment at the time of admission (from either study arm) were used to assess the extent of investigation for TB during admission.

#### Research-assigned cause of admissions.

For each recorded admission, the most likely cause of admission was assigned by a research physician (P. G. B.) using all the available information. This included information that was not available to clinicians assessing the individual in real time, such as information from PHC clinic files, results of laboratory tests conducted in other facilities, and findings from the parent trial or mortality sub-study (e.g., urine LF-LAM results or autopsy findings). Research-assigned causes were classified into mutually exclusive categories (Table 1), with criteria pre-specified for likelihood of diagnosis (possible, probable, or definite) using a standardized tool (Supplemental Table S1). System-based categories were used (renal/liver failure, and hematological, neurological, and gastrointestinal disorders) when information was insufficient to assign a more specific diagnosis.

**Table 1:** Categories for causes of admission based on prespecified criteria for likelihood of diagnosis*

Category of causes	Description of category
Previously undiagnosed TB	In individuals not on TB treatment at the point of admission with clinicopathological features in keeping with TB, with or without microbiological confirmation, and regardless of whether TB treatment was initiated during admission. Prespecified likelihood of diagnosis allocated to each ‘Previously undiagnosed TB’ admission: • Definite: microbiological evidence for TB (positive Xpert MTB/RIF assay or positive TB culture) • Probable: positive AFB microscopy; positive urine LAM; or CSF, chest X-ray, or ultrasound features in keeping with TB • Possible: clinicopathological features of TB
On TB treatment with worsening TB disease	In individuals on TB treatment admitted as a result of worsening TB disease
Bacterial infections	Admissions resulting from bacterial infections based on clinicopathological evidence for a bacterial infection, including microbiological isolation of an organism or investigation findings specific to bacterial infections. This category excludes new TB cases and surgical-related infections.
AIDS-related illnesses	Admissions secondary to WHO clinical stage IV diagnoses,^38^ excluding TB and severe bacterial infections
Treatment-related conditions	Admissions resulting from treatment side effects
Non-communicable diseases	Admissions resulting from cardiovascular diseases, diabetes, cancer (excluding AIDS-related cancers), and chronic respiratory diseases
Surgical conditions	Any surgical condition not related to another category
Psychiatric conditions	All psychiatric conditions
Renal/liver failure	All forms of renal failure and liver failure not fitting into the previous categories
Hematological disorders	Hematological conditions that do not fit into other categories
Neurological disorders	Neurological conditions that do not fit into other categories
Gastrointestinal disorder	Any gastrointestinal disorder that does not fit into other categories. This group included acute gastroenteritis with no underlying diagnosis.
Unknown	Any admission for which there was not enough recorded information in the case report form to place into any other category

AFB = acid-fast bacilli; CSF = cerebrospinal fluid; LAM = lipoarabinomannan; TB = tuberculosis.

* See Supplemental Table S1 for criteria used to assign each cause of admission.

A random sample of 20% of admissions were reviewed independently by another research physician (A. S. K.) to validate the initial research assignments. Comparison was made between causes assigned by the two reviewers, and Cohen’s κ was used to estimate inter-rater agreement. A priori, a κ value of ≥ 0.60 was considered sufficient for 1) no further validation to be conducted and 2) for the causes assigned by the primary research physician to be used for analysis.

#### Investigation of TB and treatment of TB and HIV in adults.

Evidence for the investigation of TB was sought from hospital notes and NHLS data for admissions when the individual was not on TB treatment at the point of admission. Investigations assessed were those suggested in national guidelines^20^—namely, Xpert MTB/RIF, chest radiographs, sputum microscopy, and mycobacterial blood and sputum cultures. Individuals were considered to have TB symptoms if they were recorded to have any of the four WHO screening symptoms at admission.^23^ Of the individuals started on TB treatment during admission, evidence was sought for bacteriological confirmation of TB. Treatment charts and notes were assessed to determine how many individuals were initiated on ART during admission and how many were referred to their primary-level clinic to be initiated on treatment post-discharge.

#### Analysis.

Anonymized data sets were merged by participant identification numbers; analysis was done using Stata v15 (Stata Corp., College Station, TX). For individuals with multiple admissions, data from the first admission after enrollment were used for analysis.

### Ethical considerations.

The parent trial received ethical approval from the research ethics committees of the University of the Witwatersrand, the London School of Hygiene & Tropical Medicine, the South African Medicine Controls Council, and provincial and district departments of health. This analysis was approved by the London School of Hygiene & Tropical Medicine. All participants gave informed consent, which included permission for the study team to extract information from clinical and laboratory records. No identifiers were extracted from hospital files.

## RESULTS

### Demographics.

**Figure 1.**
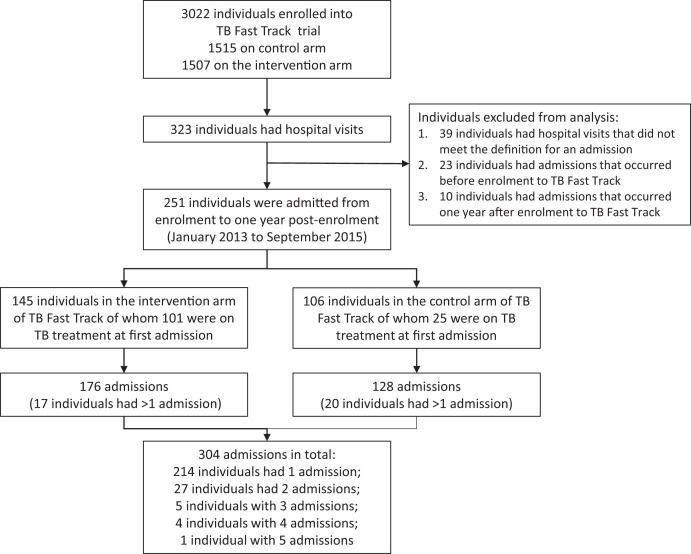
Flow diagram showing numbers of participants enrolled in the TB Fast Track trial, admitted to the hospital, included and excluded from analysis (with reasons), and the numbers of admissions recorded by study arm. TB = tuberculosis.

A total 3,022 individuals with advanced HIV were enrolled to the TB Fast track trial (1,507 in the intervention arm and 1,515 in the control arm). Overall, 285 adults had recorded hospital admissions; 34 individuals were excluded from the analysis as the admission occurred outside the specified time (Figure 1). The remaining 251 individuals [130, or 51.8%, female; median age, 38.8 years; interquartile range (IQR), 32.9–45.1 years; Table 2] experienced 304 admissions from January 2013 to September 2015 (37 individuals had more than one admission); 106 (34.9%) admissions were identified through the study team being notified and the remainder were ascertained because the individual was recorded as having died. The median time from trial enrollment to an individual’s first admission was 40 days (IQR, 14–124 days) and the median length of admission was 5 days (IQR, 2–10.5 days). A total of 101 of 145 individuals (69.7%) in the intervention arm were on TB treatment at the time of their first admission, compared with 25 of 106 individuals (23.6%) in the control arm. Although 152 of 251 individuals (60.6%) were on ART at the time of their first admission, CD4 counts at admission were less than 150 cells/µL for 108 of 114 individuals (94.7%) with a recorded result (median, 37.5 cells/µL; IQR, 14–68 cells/µL).

**Table 2:** Demographics of individuals (*N* = 251) at their first admission and by TB Fast Track trial arm

Characteristic	All individuals (*N* = 251), *n* (%) or median (IQR)	Individuals in TBFT intervention arm (*n* = 145), *n* (%) or median (IQR)	Individuals in TBFT control arm (*n* = 106), *n* (%) or median (IQR)
Female	130 (51.8)	78 (53.8)	52 (49.1)
Age (y)	38.8 (32.9–45.1)	39.3 (33.0–47.0)	37.8 (32.9–42.7)
Province			
Gauteng	192 (76.5)	113 (77.9)	79 (74.5)
Limpopo	37 (14.7)	23 (15.9)	14 (13.2)
North-West	22 (8.8)	9 (6.2)	13 (12.3)
Level of hospital admitted to			
District	238 (94.8)	142 (97.9)	96 (90.6)
Tertiary	13 (5.2)	3 (2.1)	10 (9.4)
On ART*	152 (60.6)	74 (51.0)	78 (73.6)
On TB treatment	126 (50.2)	101 (69.7)	25 (23.6)
Previous TB†	33 (13.2)	18 (12.4)	15 (14.2)
CD4 count at admission (cells/μL)			
< 50	67 (58.8)‡	39 (59.1)§	28 (58.3)‖
50–100	37 (32.5)	22 (33.3)	15 (31.3)
100–150	4 (3.5)	2 (3.0)	2 (4.2)
>150	6 (5.3)	3 (4.6)	3 (6.3)
Serum hemoglobin (g/dL)	9.2 (7.2–11.3)¶	9.2 (7.3–11.3)#	9.2 (7.2–11.4)**
Creatinine (μmol/L)	88 (65–226)††	89 (66–189)‡‡	85.5 (65–326)§§

ART = antiretroviral treatment; GFR = glomerular filtration rate; IQR = interquartile range; TB = tuberculosis; TBFT = TB Fast Track trial.

* Three individuals did not have recorded ART status.

† Previous TB prior to enrollment to the TB Fast Track trial.

‡ One hundred fourteen individuals had CD4 counts available at admission.

§ Sixty-six individuals in the intervention arm had CD4 counts available at admission.

‖ Forty-eight individuals in the control arm had CD4 counts available at admission.

¶ A total of 223 individuals had a serum hemoglobin level at the time of admission.

# A total of 127 individuals in the intervention arm had a hemoglobin level at the time of admission.

** Ninety-six individuals in the control arm had a hemoglobin level at the time of admission.

†† A total of 206 individuals had a creatinine level at the time of admission

‡‡ A total of 114 individuals in the intervention arm had a creatinine level at the time of admission.

§§ Ninety-two individuals in the control arm had a creatinine level at the time of admission.

### Research-assigned causes of admission.

Inter-rater agreement was acceptable (κ value, 0.65; 95% CI, 0.51–0.79) and causes of admission assigned by the primary research physician were used for analysis (Supplemental Table S2). TB was the most commonly assigned cause of first admission. Ninety-five of 251 individuals (37.8%) were admitted with TB [52 of 251 (20.7%) with “previously undiagnosed TB” and 43 of 251 (17.1%) with “on TB treatment with worsening TB disease”] (Table 3). “Definite” evidence was found for 20 of 95 (21.1%) TB-related admissions. The next most common research-assigned causes of first admission were AIDS-related illnesses (*n* = 41, 16.3%; Supplemental Table S3), surgical causes (*n* = 21, 8.4%), and bacterial infections (*n* = 20, 8.0%). Participant characteristics cross-referenced with research-assigned causes of admission are shown in Table 4, and a full list of participant characteristics with assigned causes and outcomes is provided in Supplemental Table S2. In subsequent admissions (second, third, and fourth admissions), AIDS-related illness was the most common research-assigned cause [14 of 53 (26.2%) admissions], followed by “on TB treatment with worsening TB disease” (*n* = 9, 17.0%) and “surgical conditions” (*n *= 8, 15.1%; Supplemental Table S4).

**Table 3:** Causes of first admission assigned according to predetermined categories and probability (*N* = 251 admissions)

Admission cause category	Total, *n* (column %)	Probability of cause,* *n* (row %)		
		Definite	Probable	Possible
Total	251	82 (32.7)	87 (34.7)	82 (32.7)
AIDS-related illness*	41 (16.3)	29 (70.7)	4 (9.8)	8 (19.5)
Previously undiagnosed TB	52 (20.7)	11 (21.2)	28 (53.9)	13 (25.0)
On TB treatment with worsening TB disease	43 (17.1)	9 (20.9)	11 (25.6)	23 (53.5)
Surgical	21 (8.4)	12 (57.1)	6 (28.6)	3 (14.3)
Treatment related	18 (7.2)	0	13 (72.2)	5 (27.8)
Bacterial infection	20 (8.0)	5 (25.0)	6 (30.0)	9 (45.0)
Non-communicable disease	14 (5.6)	10 (71.4)	2 (14.3)	2 (14.3)
Gastrointestinal disorders	12 (4.8)	0	3 (25.0)	9 (75.0)
Liver/renal failure	9 (3.6)	1 (11.1)	7 (77.8)	1 (11.1)
Neurological disorder	5 (2.0)	0	3 (60.0)	2 (40.0)
Hematological disorder	7 (2.8)	3 (42.9)	3 (42.9)	1 (14.3)
Unknown	6 (2.4)	0	0	6 (100)
Psychiatric disorder	3 (1.2)	2 (66.6)	1 (33.3)	0

TB = tuberculosis.

* See Supplemental Table S1 for criteria used to assign causes and probabilities.

**Table 4:** Selected characteristics of adults with advanced HIV admitted to hospital within 1 year of enrollment, cross-referenced with research-assigned cause of first admission (*N* = 251)

Characteristic	Total, *n* (%) or median (IQR)	Research cause assigned to first admission, *n* (column %) or median (IQR)								
		AIDS-related	Previously undiagnosed TB	Worsening TB on TB treatment	Surgical	Treatment related	Bacterial infection	Gastrointestinal	Hepatic/renal failure	Other
Total	251	41	52	43	21	18	20	12	9	35
Age group (y)										
18–23	6 (2.4)	2 (4.9)	1 (1.9)	2 (4.7)	1 (4.8)	0	0	0	0	0
24–33	70 (27.9)	9 (22.0)	21 (40.4)	11 (25.6)	7 (33.3)	7 (38.9)	2(10.0)	5 (41.7)	1 (11.1)	7 (20.0)
34–43	106 (42.2)	17 (41.5)	24 (46.2)	15 (34.9)	10 (47.6)	7 (38.9)	8 (40.0)	3 (25.0)	4 (44.4)	18 (51.4)
44–60	61 (24.3)	10 (24.4)	6 (11.5)	14 (32.6)	3 (14.3)	4 (22.2)	9 (45.0)	4 (33.3)	3 (33.3)	8 (22.9)
> 60	8 (3.2)	3 (7.3)	0	1 (2.3)	0	0	1	0	1 (11.1)	2 (5.7)
Female	130 (51.8)	21 (51.2)	27 (51.9)	18 (41.9)	17 (81.0)	12 (66.7)	8 (40.0)	4 (33.3)	4 (44.4.)	19 (54.3)
Enrolled in intervention arm of TB Fast Track trial	145 (57.8)	29 (70.7)	16 (30.8)	30(69.8)	14 (66.7)	12 (66.7)	11 (55.0)	7 (58.3)	3 (33.3)	23 (65.7)
CD4 count at enrollment to TB Fast Track trial (cells/µL)										
≤50	132 (52.6)	27 (65.9)	31 (59.6)	25 (58.1)	6 (28.6)	11 (61.1)	5 (25.0)	6 (50.0)	3 (33.3)	18 (51.4)
51–100	80 (31.9)	11 (26.8)	10 (19.2)	13 (30.2)	9 (42.9)	4 (22.2)	11 (55.0)	4 (33.3)	5 (55.6)	13 (37.1)
101–150	39 (15.5)	3 (7.3)	11 (21.2)	5 (11.6)	6 (28.6)	3 (16.7)	4 (20.0)	2 (16.7)	1 (11.1)	4 (11.4)
Time from enrollment to admission (d)										
≤ 7	30 (12.0)	6 (14.6)	9 (17.3)	8 (18.6)	0	0	0	0	0	7 (20.0)
8–28	69 (27.5)	19 (46.3)	13 (25.0)	8 (18.6)	5 (23.8)	9 (50.0)	5 (25.0)	2 (16.7)	1 (11.1)	7 (20.0)
29–90	76 (30.3)	7 (17.1)	21 (40.4)	11 (25.6)	5 (23.8)	6 (33.3)	5 (25.0)	3 (25.0)	4 (44.4)	14 (40.0)
91–180	51 (20.3)	4 (9.8)	7 (13.5)	11 (25.6)	8 (38.1)	2 (11.1)	8 (40.0)	4 (33.3)	2 (22.2)	5 (14.3)
181–365	25 (10.0)	5 (12.2)	2 (3.9)	5 (11.6)	3 (14.3)	1 (5.6)	2(10.0)	3 (25.0)	2 (22.2)	2 (5.7)
On ART at admission	152 (60.6)	17 (41.5)	34 (65.4)	26 (60.5)	17 (81.0)	9 (50.0)	13 (65.0)	10 (83.3.)	6 (66.7)	20 (57.1)
On TB treatment at admission	126 (50.2)	20 (48.8)	0	43 (100)	8 (38.1)	16 (88.9)	10 (50.0)	6 (50.0)	3 (33.3)	20 (57.1)
Creatinine and eGFR measured at admission	206	37	45	37	14	14	17	7	9	26
Creatinine level at admission (µmol/L)	88 (65–226)	77 (65–114)	105 (75–264)	88 (65–189)	74 (56–98)	93 (68–134)	106 (68–503)	118 (63–393)	1,324 (1,018– 1,606)	75 (58–100)
eGFR at admission (mL/min/1.73 m^2^)										
≤ 30	60 (29.1)	8 (21.6)	14 (31.1)	11 (29.7)	2 (14.3)	3 (21.4)	7 (41.2)	3 (42.9)	9 (100.0)	3 (11.5)
31–60	25 (12.1)	3 (8.1)	8 (17.8)	4 (10.8)	2 (14.3)	3 (21.4)	2 (11.7)	1 (14.3)	0	2 (7.7)
61–90	37 (18.0)	6 (16.2)	9 (20.0)	6 (16.2)	4 (28.6)	3 (21.4)	3 (17.6)	1 (14.3)	0	5 (19.2)
> 90	84 (40.8)	20 (54.1)	14 (31.1)	16 (43.2)	6 (42.9)	5 (35.7)	5 (29.4)	2 (28.6)	0	16 (61.5)
Hb measured at admission	223	35	49	40	17	16	17	11	9	29
Hb at admission (g/dL)										
< 7.0	51 (22.8)	5 (14.3)	15 (30.6)	6 (15.0)	2 (11.8)	1 (6.3)	8 (47.1)	1 (9.1)	4 (44.4)	9 (31.0)
7.0–8.9	50 (22.4)	8 (22.9)	12 (24.5)	13 (32.5)	3 (17.7)	1 (6.3)	5 (29.4)	3 (27.3)	3 (33.3)	2 (6.9)
9.0–10.9	56 (25.1)	9 (25.7)	9 (18.4)	11(27.5)	4 (23.5)	6 (37.5)	4 (23.5)	3 (27.3)	1 (11.1)	9 (31.0)
11.0–12.9	47 (21.1)	12 (34.3)	8 (16.3)	9 (22.5)	6 (35.3)	6 (37.5)	0	2 (18.2)	1 (11.1)	3 (10.3)
≥ 13.0	19 (8.5)	1	5 (10.2)	1 (2.5)	2 (11.8)	2 (12.5)	0	2 (18.2)	0	6 (20.7)

ART = antiretroviral therapy; eGFR = estimated glomerular filtration rate; Hb = hemoglobin; IQR = interquartile range; TB = tuberculosis.

Unsurprisingly, given the nature of the intervention, more participants in the control arm than the intervention arm of TB Fast Track were assigned a “previously undiagnosed TB” admission cause (control: 38 of 128, 29.7%; intervention: 17 of 176, 9.7%), and more individuals in the intervention arm than the control arm were assigned a “worsening TB disease on TB treatment” admission cause (control: 17 of 128, 13.3%; intervention: 35 of 176, 19.9%). Ninety-one participants enrolled to the TB Fast Track intervention group who were assessed to have a high probability of TB based on the trial’s algorithm (LF-LAM positive, hemoglobin < 10 g/dL, or body mass index < 18.5 kg/m^2^) were admitted. Twenty-five of 91 participants (27.5%) were assigned a cause of “worsening TB disease on TB treatment” and 18 of 91 participants (19.8%) were assigned a cause of “AIDS-related illness” (Supplemental Table S5).

### Outcomes of admissions.

**Figure 2.**
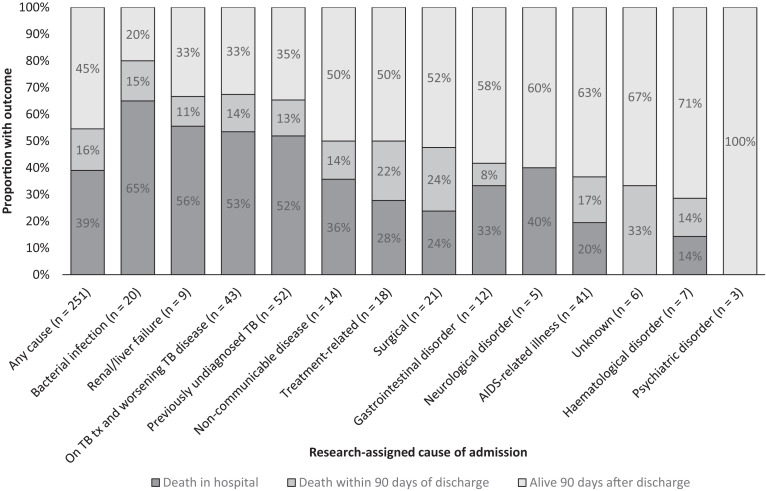
Outcome of first admission by cause of admission (*N* = 251). TB = tuberculosis; tx = treatment.

Ninety-eight of 251 individuals (39.0%) died in the hospital during their first admission; 151 of 251 individuals (60.2%) were discharged home (two individuals were transferred to different facilities and the outcomes are unknown). Of the 151 who were discharged, 39 (25.8%) died within 90 days of discharge and 19 (12.6%) were readmitted to hospital within 30 days of discharge (Supplemental Table S6). Fifteen individuals died during subsequent admissions, bringing overall in-patient mortality to 113 of 251 (45.0%). A total 137 of 251 individuals (54.6%) died in the hospital or 90 days after discharge from their first admission (Figure 2). Of the 95 individuals with a research-assigned TB-related cause of first admission, 50 (52.6%) died during admission and a further 13 (13.7%) died within 90 days post-discharge. Of the 52 individuals assigned a “previously undiagnosed TB” cause of admission, 12 of 22 (54.5%) who did not receive anti-TB treatment died in the hospital, and 15 of 30 individuals (50.0%) who did receive anti-TB treatment died as inpatients.

### TB symptoms and investigation for TB.

**Figure 3.**
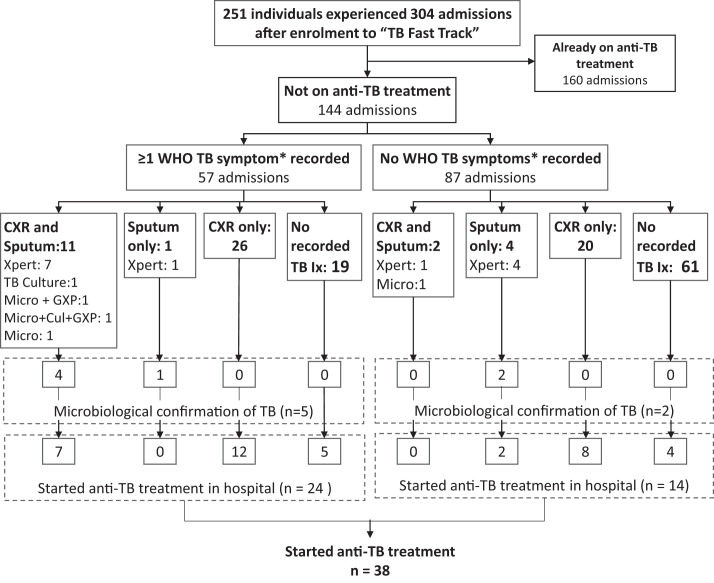
Tuberculosis (TB) diagnostics performed on admissions not on anti-TB treatment (*n* = 144) comparing symptomatic (*n* = 57) and asymptomatic (*n* = 87) admissions. Cul = TB culture; CXR = chest X-ray; GXP = Xpert MTB/RIF assay; Ix = investigations; micro = microscopy; Xpert = Xpert MTB/RIF assay (GXP and Xpert represent the same test). * Cough, fever, night sweats, or weight loss.

Of the total 304 admissions, 144 (47.4%) occurred in individuals who were not on anti-TB treatment at the point of admission; recorded TB symptoms, investigations, and treatment were examined in these 144 admissions (Figure 3). At least one TB symptom was recorded in 57 of 144 admissions (39.6%): cough in 43 of 144 (29.9%), weight loss in 28 of 144 (19.4%), fever in 21 of 144 (14.6%) and night sweats in 14 of 144 (9.7%). At least one investigation for TB was conducted in 64 of 144 admissions (44.4%); a sputum sample was collected in 18 of 144 individuals (12.5%) and a chest radiograph was conducted in 59 of 144 individuals (41.0%). Twenty-one sputum tests were conducted on the 18 sputum samples collected. Positive results were seen in one of four sputum samples (25.0%) sent for TB microscopy, 6 of 15 (40.0%) sent for Xpert MTB/RIF testing; and in one of two (50.0%) sent for mycobacterial culture. No mycobacterial blood cultures were performed. Of the 57 admissions for whom at least one WHO TB symptom was documented, sputum was collected 12 of 57 times (21.1%), and a chest radiograph was conducted 37 of 57 times (64.9%). Selected characteristics for those who had sputum collected or had a chest radiograph while hospitalized are described in Supplemental Table S7.

### Management of TB and HIV as an inpatient.

In 102 of 304 admissions (33.6%), the individual was not on ART at the time of admission. In 7 of 102 admissions (6.9%), ART was initiated as an in-patient. In 48 of 102 admissions (47.1%), ART was deferred because there were concomitant reasons not to initiate ART. However, in 47 of 102 admissions (46.1%), ART was not initiated and potential reasons for deferral were not recorded. In 19 of 47 of these instances (40.4%), the individual was referred to a local clinic to start ART.

Anti-TB treatment was initiated during 38 admissions. In 24 of 38 admissions (63.2%), one or more WHO TB symptom had been recorded, and in 9 of 38 admissions (22.5%) a sputum-based investigation had been conducted. Empirical anti-TB treatment (without a positive sputum result) was initiated on 32 of 38 occasions (84.2%).

## DISCUSSION

In the context of a pragmatic trial that recruited ambulatory adults with advanced HIV attending PHC clinics in South Africa, we used a wide range of data and a structured and transparent process to examine 304 admissions in 251 individuals, and described causes for admission, mortality, and investigation for TB. More than one third of admissions were TB related. Overall mortality was very high: 55% of individuals in this analysis (median age, 39 years) died in hospital or within 90 days of discharge from their first admission. Of those admitted with TB, bacterial infections, or renal or liver failure, the proportion who died was 65% or more. TB was generally under-investigated. Sputum-based investigations were used in only 21% of those with at least one recorded WHO TB symptom. Our findings underline the need for improvements along the care pathway for adults with advanced HIV, from community clinics and in-hospital management to post-discharge follow-up, to ensure more timely referral to secondary care, greater awareness and appropriate investigation for TB among inpatients, and prompt initiation of ART.^24^

Our most striking finding is the high mortality rate associated with admission. All those included in this analysis attended a PHC clinic and were identified as HIV-positive a median of 40 days prior to admission, which presented a window of opportunity for clinical support. The WHO recommends a “package of interventions” to reduce HIV-associated morbidity and mortality that includes the need for early identification of HIV,^1^ screening for opportunistic infections, appropriate prophylaxis, and initiation of ART through a test-and-treat strategy.^25^ Prompt ART initiation was strongly encouraged in the intervention arm of this pragmatic trial, however clinical decisions were made by clinic staff. In our analysis, only 60% of individuals were on ART at the time of their first admission, and ART was initiated as an inpatient for seven others. Along with strategies to improve access to care and expand HIV testing,^26^ more regular engagement with patients by PHC clinics during and after ART initiation may be beneficial in detecting warning signs and enabling earlier referral to a hospital.

The post-discharge period is also high risk. Other studies in South Africa have shown that mortality at 90 days post-discharge for HIV-positive adults ranges from 13% to 26%.^27^^,^^28^ In our analysis, 26% of individuals died within 90 days of their first discharge (15 as inpatients during a subsequent admission), and 13% of those discharged were readmitted within 30 days. Strengthening processes for ensuring clear communication and handover to primary or community care may help to reduce readmission rates and improve outcomes.^29^

Our study shows that an important part of in-hospital management of adults with advanced HIV relies on having a high level of suspicion for infection-related illnesses (TB, bacterial, and other AIDS-related illnesses), which are still the most common causes of admission for HIV-positive adults in this setting.^3^^,^^10^^,^^27^ We estimated that one third of admissions in our analysis were TB related. TB has been shown to account for 18% to 33% of hospital admissions among HIV-positive adults in similar settings.^10^^,^^14^^,^^27^ Even with a high suspicion for TB, making a timely diagnosis is difficult because of the limitations of the available investigations, many of which are less sensitive in people with low CD4 counts. However, we found that even the use of TB investigations was limited. Sputum tests for TB were performed in only 12.5% of admissions, regardless of presenting complaint, and in 21.1% of those who reported one or more WHO TB symptom. Others have shown that TB sputum tests may be underused because very ill HIV-positive adults are unable to produce sputum as a result of their illness,^14^ because clinicians may not suspect TB as a result of the atypical presentation of the disease in adults with advanced HIV,^14^ and because clinicians in these settings may see little advantage in performing sputum investigations when the diagnosis can be made clinically or radiologically.^30^^,^^31^ However, despite the reduced sensitivity of sputum Xpert MTB/RIF testing in HIV-positive adults,^32^ part of its utility at the population level comes from its consistent use in those at high risk of TB. Therefore, the underuse documented in this study represents a missed opportunity to maximize the overall number of TB diagnoses made.

Disseminated TB in adults with advanced HIV may also be missed by an algorithm that is reliant on sputum investigations.^33^ A recent meta-analysis found that the detection of TB bloodstream infection in adults with advanced HIV increased from 77% (95% CI, 63–87%) when only sputum investigations were used, to 89% (95% CI, 80–94%) when used in combination with the urine LAM test.^34^ The use of urine LAM with subsequent initiation of anti-TB treatment has been shown to reduce mortality in hospitalized adults with advanced HIV,^35^ although its inability to detect drug-resistant TB may limit its utility.^36^ Because of the high prevalence of TB in this population, and the extremely high mortality associated with it,^37^ intensive use of imaging and all available TB tests on both respiratory and non-respiratory specimens may be needed in all hospitalized adults with advanced HIV regardless of presenting symptoms.^14^^,^^34^ Furthermore, as autopsy studies have demonstrated, multiple concomitant pathologies may be present along with TB. Packages of care for these individuals should include appropriate investigation, prophylaxis, and treatment of other infections and malignancies in addition to TB.

This article considers only a small proportion of the individuals enrolled in the TB Fast Track trial, but our findings may help explain the results of the trial, which found that the use of empirical TB treatment, guided by a novel, point-of-care algorithm, did not reduce mortality in ambulatory adults with advanced HIV. Our analysis shows the variety of conditions, including bacterial infections and AIDS-defining illnesses, that contribute to morbidity and mortality in this group. It is worth noting that among those in this analysis with “previously undiagnosed TB,” mortality was around 50% for those who were not started on TB treatment as inpatients, but was also around 50% for those who were started on TB treatment. Identifying and treating TB, although clearly important, is unlikely to be the only intervention needed in people with advanced HIV disease. Pragmatic trials of alternative strategies, including packages of care and systems-level interventions to identify and engage people earlier in the illness trajectory,^38^ are urgently needed to reduce the numbers of preventable deaths seen in these vulnerable individuals.

### Limitations and strengths.

Our analysis was based on a single reviewer’s categorization of admissions; but, to delineate the quality of evidence and likelihood of diagnosis, a probability was allocated for each admission, and 20% of cases were blind-reviewed to validate the initial allocations. This was also a retrospective study. When allocating causes of admission, there was a reliance on the investigations performed, accurate documentation of symptoms and signs, and attending clinicians’ interpretations of imaging. Gaps in the available data meant that a high proportion (66%) of research-assigned causes of admission were assigned with only a “possible” or “probable” likelihood; and broad, system-based “syndromes” were used when information was not sufficient even to estimate a more specific category. Our findings and conclusions should be interpreted in the context of this uncertainty. Only the most likely cause of admission was assigned for each admission, which may have underestimated the proportion of individuals with multiple pathology at the time of admission. In the TB Fast Track autopsy sub-study (*n* = 34), around 60% of those autopsied had evidence of two or more concurrent infections.^39^ These results also need to be considered in the context of the parent trial;^18^ the intervention increased the number of persons admitted on TB treatment, which would have decreased the number of admissions with previously undiagnosed TB and underestimated the extent to which TB treatment was initiated in the hospital. Hospitalization was also likely under-ascertained in control clinics because fewer study visits were required and participants likely had less interaction with research and clinic staff. Admissions included in this analysis took place between 2013 and 2015 and may not be reflective of current practice in the hospitals included. However, South African national guidelines for the investigation and treatment of TB have not changed substantially in the intervening time,^20^ and studies reporting more recent data also show considerable TB-related morbidity and mortality in hospitalized individuals with advanced HIV. Two thirds of admissions were identified because the individual was known to have died, which may have led to overestimation of the overall case fatality rate. This study’s strengths include the examination of the course of illness and clinical management of a well-defined group of individuals for the full length of admission, and use of a transparent and reproducible method to assign causes of admission, linking these to high-quality information on mortality.

## CONCLUSION

Hospitalized adults with advanced HIV are at high risk of death. TB was a common cause of hospitalization but was under-investigated, even in those with symptoms. In addition to early identification of TB and other AIDS-related illnesses during hospitalization of adults with advanced HIV, improved pre-hospital management strategies are needed to interrupt disease progression and reduce poor outcomes in this already vulnerable population.

## Supplemental Material


Supplemental materials
