# Supplementary material for: Causes and Outcomes of Admission and Investigation of Tuberculosis in Adults with Advanced HIV in South African Hospitals: Data from the TB Fast Track Trial
Source: Am J Trop Med Hyg. 2021 Oct 18;105(6):1662–71. doi: 10.4269/ajtmh.21-0133 (PMC8641325; doi:10.4269/ajtmh.21-0133)
Supplement: Supplementary file 1 [file tpmd210133.SD1.pdf]

Supplementary table 1: Research-assigned causes of admission: tool and criteria for probability categories

Cause of admission study category	Description of admission category	Likelihood of diagnosis based on Evidence		
		**Definite**	**Probable**	**Possible**
**Previously undiagnosed TB**	All types of TB (individual cannot be on TB treatment at admission)	Documented history and clinicopathological features in keeping with active TB disease<br>AND<br>Microbiological evidence of TB (Xpert MTB/RIF positive; culture positive) from any site.	Documented history and clinicopathological features suggestive of tuberculosis disease<br>AND<br>Documentation of<br>Chest X-ray, cerebrospinal fluid analysis; or ultrasonographic features in keeping with TB<br>OR<br>Positive AFB microscopy	History and clinicopathological findings in keeping with tuberculosis disease<br><br>History may include weight loss, chronic cough, fever and night sweats.
**On TB treatment admitted with worsening TB symptoms**	Individual admitted on TB treatment with clinical deterioration secondary to tuberculosis	Documentation of TB treatment<br>AND<br>History and clinicopathological evidence in keeping with tuberculosis disease<br>AND<br>Confirmed mycobacterium tuberculosis on culture or Xpert MTB/RIF prior or during admission.	Documentation of TB treatment<br>AND<br>History and clinicopathological evidence of worsening tuberculosis disease<br>AND<br>Chest X-ray; ultrasonographic features; or cerebral fluid analysis in keeping with TB<br>OR<br>Positive AFB Microscopy prior or during admission.	Documentation of TB treatment<br>AND<br>History and clinicopathological evidence of worsening tuberculosis disease
**Bacterial infection**	Bacterial pneumonia<br>Bacterial meningitis<br>Urinary tract infections<br>Pelvic inflammatory disease<br>Pyelonephritis<br>Dysentery<br>Unspecified septicaemia<br>(*Excluding TB and bacterial infections associated with surgical conditions*)	Microbiological isolation of an organism<br>AND<br>Documented history and clinicopathological evidence in keeping with condition	Documented history and clinicopathological evidence in keeping with bacterial infection<br>AND<br>Investigations specific to focus of infection.<br>For example, documented changes on X-ray in keeping with pneumonia or cerebral fluid analysis in keeping with bacterial meningitis.	Documented history and clinicopathological evidence in keeping with a bacterial infection.

Cause of admission study category	Description of admission category	Likelihood of diagnosis based on Evidence		
		**Definite**	**Probable**	**Possible**
**AIDS-related illnesses**	All AIDS-related illnesses [1]<br><br>(*excluding tuberculosis, bacterial infections, and symptomatic HIV-associated nephropathy*)	Isolation of organism or a positive specific diagnostic test to determine the following AIDS related illnesses:<br>o HIV wasting syndrome<br>o HIV encephalopathy<br>o Disseminated non-tuberculosis mycobacteria infection<br>o Progressive multifocal leucoencephalopathy<br>o Cryptosporidiosis<br>o Isosporiasis<br>o Visceral herpes simplex infection<br>o Cytomegalovirus infection<br>o Any disseminated mycosis<br>o Lymphoma (cerebral or B-cell non-Hodgkin)<br>o Invasive cervical carcinoma<br>o Kaposi Sarcoma<br>o Oesophageal candidiasis<br>o Pneumocystis pneumonia<br>o Extrapulmonary cryptococcosis including meningitis	History and clinicopathological evidence in keeping with AIDS related illnesses<br>AND<br>Documented laboratory or imaging findings in keeping with illness but not specific enough to give a definite diagnosis(e.g., X-ray findings in keeping with PCP) to determine the following AIDS-related illnesses:<br>o HIV wasting syndrome<br>o HIV encephalopathy<br>o Disseminated non-tuberculosis mycobacteria infection<br>o Progressive multifocal leucoencephalopathy<br>o Cryptosporidiosis<br>o Isosporiasis<br>o Visceral herpes simplex infection<br>o Cytomegalovirus infection<br>o Any disseminated mycosis<br>o Lymphoma (cerebral or B-cell non-Hodgkin)<br>o Invasive cervical carcinoma<br>o Kaposi Sarcoma<br>o Oesophageal candidiasis<br>o Pneumocystis pneumonia<br>o Extrapulmonary cryptococcosis including meningitis	Documented history and clinicopathological evidence in keeping with the following AIDS related illnesses:<br>o HIV encephalopathy<br>o HIV wasting syndrome<br>o Disseminated non-tuberculosis mycobacteria infection<br>o Progressive multifocal leucoencephalopathy<br>o Cryptosporidiosis<br>o Isosporiasis<br>o Visceral herpes simplex infection<br>o Cytomegalovirus infection<br>o Any disseminated mycosis<br>o Lymphoma (cerebral or B-cell non-Hodgkin)<br>o Invasive cervical carcinoma<br>o Kaposi Sarcoma<br>o Oesophageal candidiasis<br>o Pneumocystis pneumonia<br>o Extrapulmonary cryptococcosis including meningitis

Cause of admission study category	Description of admission category	Likelihood of diagnosis based on Evidence		
		**Definite**	**Probable**	**Possible**
**Treatment-related conditions**	Drug induced liver injury [2]	Documentation that individual is on TB, ART or co-trimoxazole treatment<br>AND<br>ALT level > 120 IU/L and symptomatic (nausea, vomiting, abdominal pain, jaundice)<br>OR<br>ALT level >200 IU/L and asymptomatic or Total serum bilirubin concentration > 40µmol/l<br>AND<br>Other causes including pre-existing liver disease, viral hepatitis, TB involvement of the liver, IRIS and bacterial sepsis have been excluded.	Documentation that individual on TB, ART or cotrimoxazole treatment<br>AND<br>ALT level >120 IU/L and symptomatic (nausea, vomiting, abdominal pain, jaundice)<br>OR<br>ALT level >200 IU/l and asymptomatic or Total serum bilirubin concentration >40 µmol/l<br>AND<br>More likely to be attributable to the drug despite other causes not being excluded	Documentation that history and clinicopathological evidence in keeping with worsening function following the introduction of the treatment
	Other treatment-related conditions	Documentation that individual on treatment<br>AND<br>Documented history and clinicopathological evidence in keeping with worsening function following the introduction of the drug<br>AND<br>Diagnostics have been done to exclude other causes for the syndrome	Documentation that individual on treatment<br>AND<br>documented history and clinicopathological evidence in keeping with worsening function on the introduction of the drug<br>AND<br>More likely to be attributable to the drug despite other causes not being excluded.	Documentation that history and clinicopathological evidence in keeping with worsening function following the introduction of the treatment
**Non-communicable disease**	Cardiovascular diseases; diabetes; non-AIDs cancers; and chronic respiratory diseases	Documented diagnostics that are specific for condition<br>AND<br>Documented history and clinicopathological evidence in keeping with condition.	Documented history and clinicopathological evidence in keeping with condition.	Documented history in keeping with condition.

Cause of admission study category	Description of admission category	Likelihood of diagnosis based on Evidence		
		**Definite**	**Probable**	**Possible**
**Surgical condition**	Any surgical condition including gynaecological, neurosurgical, obstetric, and cardiothoracic conditions not related to HIV or TB. Includes conditions that would be treated in a surgical ward. (*Excluding non-AIDS and AIDS-related cancers*)	Surgical intervention, imaging, or histopathological evidence specific to surgical condition<br>AND<br>Documented history and clinicopathological evidence in keeping with condition	Documented history and clinicopathological evidence in keeping with surgical condition.	Documented history in keeping with surgical condition.
**Psychiatric conditions**	Psychiatric conditions including depression, parasuicides, and psychotic related illnesses	Documented history and clinicopathological evidence in keeping with condition<br>AND<br>Laboratory evidence including LP or CT scan excluding organic causes	Documented history and clinicopathological evidence in keeping with condition<br>AND<br>Some laboratory evidence of excluding organic causes but no LP or CT scan	Documented history and clinicopathological evidence in keeping with condition
**End organ failure (renal or liver failure)**	Renal or liver failure in patients that cannot be placed in categories above and the symptoms of failure are the main reason for admission.	A documented biopsy showing evidence of renal disease or liver disease causing end organ failure<br>AND<br>Documented history and clinicopathological features in keeping with acute or chronic renal failure or other renal disorder.	Documented history and clinicopathological features in keeping with renal or hepatic failure	Documented history in keeping with renal or liver failure.
**Haematological syndrome**	Any haematological disorder not fitting into above criteria<br><br>(*Including admissions presenting with symptoms caused by haematological abnormalities including anaemia, bicytopaenia and pancytopaenia where there was no documented evidence to place the admission into another category*)	A positive test specific to haematological disorder<br>AND<br>Documented history and clinicopathological findings in keeping with haematological disorder	Documented history and clinicopathological findings in keeping with haematological disorder	Documented history in keeping with a neurological condition.

Cause of admission study category	Description of admission category	Likelihood of diagnosis based on Evidence		
		**Definite**	**Probable**	**Possible**
**Neurological syndrome**	Any neurological disorder not fitting into above categories (AIDS, Bacterial, Surgical, TB, other infections)<br><br>(*Including cases where neurological symptoms are present but the underlying cause for the symptoms are unclear.*)	A positive test specific to neurological disorder<br>AND<br>Documented history and clinicopathological findings in keeping with haematological disorder	Documented history, clinicopathological features and imaging in keeping with a neurological disorder	Documented history in keeping with a neurological condition.
**Diarrhoeal / GIT syndrome**	Any gastrointestinal disorder including diarrhoea not fitting into the above categories<br><br>(*including admissions where the underlying cause of the diarrhoea is unknown*)<br><br>Chronic diarrhoea of greater than 4 weeks will be considered an AIDS-related illness	A positive test for a specific diarrhoeal/GIT disorder<br>AND<br>Documented history and clinicopathological features in keeping with diarrhoeal/GIT disorder	Documented history and clinicopathological features in keeping with diarrhoeal disease	Documented history in keeping with a gastrointestinal condition
**Unknown cause**				Not enough information recorded to assign a cause

AFB: acid-fast bacilli; AIDS: acquired immune deficiency syndrome; ALT: alanine aminotransferase; ART: antiretroviral therapy; COPD: chronic obstructive pulmonary disease; CT: computed tomography; GIT: gastrointestinal tract; IRIS: immune reconstitution inflammatory syndrome; LP: lumbar puncture; PCP: pneumocystis pneumonia; TB: tuberculosis

1. World Health Organization. HIV / AIDS Programme WHO case definitions of HIV for surveillance and revised clinical staging and immunological classification of HIV-related diseases. **2006**; :9–22.
2. Jong E, Conradie F, Berhanu R, et al. Consensus statement: Management of drug-induced liver injury in HIV-positive patients treated for TB. South Afr J HIV Med **2013**; 14:113.

Supplementary table 2: Demographics, causes of admission, and outcomes of every individual included in the analysis (n = 251)

Pt No	TB Fast Track trial arm	Days from enrolment to admission	Admission order	Presenting problem	Duration of admission (days)	Hospital level	Age (yrs)	Sex	On ART	On TB tx	HB (g/dl)	CD4 (cells/ µL)	Creatinine µmol/L	Assigned cause of admission by primary physician	Assigned cause of admission by secondary physician	Likelihood of cause of admission	Outcome	Died within 90 days of discharge
1	Intervention	59	First	Cough for 2 weeks	2	District	24	F	N	N	11	70	54	Previously undiagnosed TB		Probable	Discharged home	Yes
2	Intervention	123	First	General weakness, poor appetite, headache	2	District	45	M	Y	Y	10.1		55	Bacterial infection		Possible	Discharged home	Yes
3	Intervention	172	First	Headache and vomiting	5	District	51	M	N	N	10		48	Bacterial infection		Definite	Discharged home	
4	Intervention	56	First	General body weakness and shivering for 1 day	4	District	30	F	Y	Y	8.4		31	On TB treatment with worsening TB disease		Definite	Death	
5	Intervention	10	First	Talking to herself and hallucinating 9 days after starting ART	36	District	33	F	Y	Y				Treatment-related		Possible	Death	
6	Intervention	106	First	Diarrhoea, fever and night sweats for 8 weeks	6	District	35	F	N	N	8.7		49	Previously undiagnosed TB		Probable	Discharged home	
7	Intervention	33	First	Diarrhoea, vomiting and weakness	4	District	46	M	Y	Y	9			Gastrointestinal		Possible	Discharged home	
8	Intervention	170	First	Body weakness and vomiting for 10 days	11	District	37	M	Y	Y	9		58	On TB treatment with worsening TB disease	AIDS-related illness	Possible	Death	
9	Intervention	14	First	Itchy rash	1	District	34	F	N	Y	11.4		59	Treatment-related		Possible	Discharged home	
10	Intervention	355	First	Body weakness, weight loss, fever for 17 days	5	District	28	M	Y	Y	4.7		632	On TB treatment with worsening TB disease		Possible	Death	
11	Intervention	169	First	Diarrhoea and vomiting	1	District	31	M	Y	Y	10.1		47	Gastrointestinal	Gastrointestinal	Possible	Discharged home	
12	Intervention	174	First	General body weakness, Loss of appetite, diarrhoea, and coughing	4	District	46	F	N	Y	8.9		38	On TB treatment with worsening TB disease		Possible	Discharged home	
13	Intervention	45	First	Body weakness, diarrhoea and vomiting	7	District	31	F	Y	Y	3.1		43	AIDS related illness		Possible	Discharged home	Yes

Pt No	TB Fast Track trial arm	Days from enrolment to admission	Admission order	Presenting problem	Duration of admission (days)	Hospital level	Age (yrs)	Sex	On ART	On TB tx	HB (g/dl)	CD4 (cells/ µL)	Creatinine µmol/L	Assigned cause of admission by primary physician	Assigned cause of admission by secondary physician	Likelihood of cause of admission	Outcome	Died within 90 days of discharge
13	Intervention	55	Second	Severe diarrhoea and vomiting	1	District	31	F	Y	Y				AIDS related illness	Bacterial infection	Possible	Death	
14	Intervention	226	First	Diarrhoea and vomiting	4	District	33	F	Y	N	8.6			Gastrointestinal		Possible	Death	
15	Intervention	28	First	Headache, vomiting, weight loss, night sweats, fever	10	District	37	M	Y	Y				On TB treatment with worsening TB disease		Definite	Discharged home	Yes
16	Intervention	29	First	Uncontrolled blood pressure	1	District	56	F	N	Y				Non-communicable		Definite	Discharged home	
17	Intervention	14	First	Shortness of breath and dry cough for 1 day	0	District	40	M	N	Y				Bacterial infection		Probable	Death	
18	Intervention	245	First	General body weakness, stomach pain and diarrhoea	4	District	49	F	Y	Y	7.7		189	On TB treatment with worsening TB disease		Possible	Death	
19	Control	81	First	Decreased level of consciousness	3	District	43	F	Y	N	7.7		56	Bacterial infection		Possible	Death	
20	Control	5	First	Diarrhoea and vomiting for weeks; loss of appetite, weight loss and night sweats	2	District	32	F	Y	N	11		44	Previously undiagnosed TB		Probable	Discharged home	
21	Control	64	First	Haemoptysis; body weakness, loss of appetite, cough	6	District	44	M	Y	N		2		Previously undiagnosed TB		Definite	Death	
22	Control	49	First	Jaundice, severe body pains, general malaise, cough and vomiting	18	Tertiary	43	M	Y	Y	9	11	55	On TB treatment with worsening TB disease		Definite	Death	
23	Control	62	First	Faeces coming out of anal fistula	30	Tertiary	34	F	Y	N	7.2	2	70	Surgical		Definite	Death	
24	Control	56	First	Chest pain, vomiting, loss of appetite, general body weakness for 2 days	0	Tertiary	38	F	Y	N		9		Previously undiagnosed TB		Probable	Death	

Pt No	TB Fast Track trial arm	Days from enrolment to admission	Admission order	Presenting problem	Duration of admission (days)	Hospital level	Age (yrs)	Sex	On ART	On TB tx	HB (g/dl)	CD4 (cells/ µL)	Creatinine µmol/L	Assigned cause of admission by primary physician	Assigned cause of admission by secondary physician	Likelihood of cause of admission	Outcome	Died within 90 days of discharge
25	Control	57	First	Severe jaundice	8	Tertiary	41	M	N	Y	13.2	74	78	Treatment-related		Possible	Discharged home	
25	Control	111	Second	Admission for worsening liver functions	29	Tertiary	41	M	Y	Y	11.6		81	Treatment-related		Probable	Discharged home	Yes
25	Control	177	Third	Cough, chest pain, shortness of breath, weakness for a 1 weeks	5	Tertiary	41	M	Y	Y	13.5		109	AIDS related illness		Probable	Death	
26	Control	135	First	Diarrhoea and general body weakness for 2 weeks.	10	Tertiary	33	M	Y	Y	7.9	121	27	On TB treatment with worsening TB disease		Probable	Discharged home	Yes
27	Intervention	116	First	Diarrhoea, cough, nose bleed and bedridden for 1 month	3	Tertiary	32	F	Y	N	2.9			Haematological		Probable	Death	
28	Intervention	31	First	General body weakness, short of breath, confusion for 1 week	0	Tertiary	35	F	Y	N	2.8	13		Neurological		Possible	Death	
29	Intervention	10	First	Productive cough, pleuritic chest pain, night sweats, loss of weight for 2 weeks	1	Tertiary	26	F	Y	Y	6.8	157		On TB treatment with worsening TB disease		Definite	Death	
30	Control	172	First	General body weakness, dyspnoea, diarrhoea, headache and neck stiffness for 2 weeks	0	Tertiary	39	F	Y	N		19		Bacterial infection		Definite	Death	
31	Control	16	First	Cough, vomiting, loss of weight, shortness of breath for 3 weeks	17	Tertiary	41	M	Y	N	8.4	58	85	Previously undiagnosed TB		Definite	Discharged home	Yes
32	Control	58	First	Collapse, shortness of breath, cough, nose bleed for 1	1	Tertiary	30	F	Y	N	3.7		864	Previously undiagnosed TB		Probable	Death	

Pt No	TB Fast Track trial arm	Days from enrolment to admission	Admission order	Presenting problem	Duration of admission (days)	Hospital level	Age (yrs)	Sex	On ART	On TB tx	HB (g/dl)	CD4 (cells/ µL)	Creatinine µmol/L	Assigned cause of admission by primary physician	Assigned cause of admission by secondary physician	Likelihood of cause of admission	Outcome	Died within 90 days of discharge
				week														
33	Control	34	First	Cough, vomiting and diarrhoea, general body pain for 2 months	1	Tertiary	46	M	Y	N	17.5	44	748	Previously undiagnosed TB		Probable	Death	
34	Control	66	First	Headache, neck stiffness and hand weakness for 1 week	7	Tertiary	47	F	Y	N	9.3	180	47	Neurological		Probable	Discharged home	
34	Control	319	Second	Neck stiffness, headache and photophobia	6	Tertiary	47	F	Y	N	12.9		59	Neurological		Possible	Discharged home	
35	Control	12	First	Weakness, dizziness and dry cough	2	District	27	M	Y	N	8.9		86	Previously undiagnosed TB		Possible	Discharged home	
35	Control	32	Second	Vomiting and general body weakness	1	District	27	M	Y	Y				Treatment-related		Probable	Discharged home	
35	Control	79	Third	Diarrhoea and abdominal pain	12	District	27	M	Y	Y	9.3		425	AIDS related illness		Possible	Discharged home	
36	Control	30	First	Productive cough, weight loss, fever and night sweats for 2 weeks	2	District	32	F	N	N	9.4		58	Previously undiagnosed TB		Possible	Discharged home	
37	Control	315	First	Shortness of breath, general body pains, vomiting and diarrhoea for 3 weeks	5	District	41	M	Y	N	9.5	8	61	Previously undiagnosed TB		Possible	Discharged home	Yes
38	Control	11	First	Difficulty in breathing for 1 day; loss of weight and cough	0	District	41	M	N	N	9.1		503	Bacterial infections		Possible	Death	
39	Control	9	First	Generalised body weakness, painful feet and cough	33	District	42	M	N	N	13.1	68	75	Previously undiagnosed TB		Probable	Death	
40	Control	94	First	Productive cough, chest pain, loss of weight and appetite, night	1	District	43	M	Y	N	11.3		401	Previously undiagnosed TB		Probable	Discharged home	Yes

Pt No	TB Fast Track trial arm	Days from enrolment to admission	Admission order	Presenting problem	Duration of admission (days)	Hospital level	Age (yrs)	Sex	On ART	On TB tx	HB (g/dl)	CD4 (cells/ µL)	Creatinine µmol/L	Assigned cause of admission by primary physician	Assigned cause of admission by secondary physician	Likelihood of cause of admission	Outcome	Died within 90 days of discharge
				sweats														
41	Control	12	First	Cough for 2 weeks; vomiting and general weakness for 1 week	2	District	42	M	Y	N	8.3		410	Previously undiagnosed TB		Probable	Death	
42	Control	86	First	Pregnant at 31 weeks with high blood pressure and fitting	12	District	36	F	Y	N	14.9		72	Surgical		Definite	transferred to another facility	
43	Control	44	First	Vomiting , general body weakness and weight loss for 1 week	5	District	33	F	Y	N	3	46	880	Previously undiagnosed TB		Definite	Death	
44	Control	52	First	Difficulty in talking, general body pains	1	District	39	M	Y	N	8.4		1436	AIDS related illness		Probable	Death	
45	Control	156	First	Foul smelling discharge for more than 1 year	11	District	39	M	Y	N	11.4	239	58	AIDS related illness		Definite	Discharged home	
46	Control	7	First	Severe headache	4	District	44	M	N	N	10.5	42	81	Previously undiagnosed TB		Definite	Discharged home	
46	Control	146	Second	Shortness of breath, night sweats, and productive cough for 1 week	6	District	45	M	Y	Y	8.8	32	119	On TB treatment with worsening TB disease		Definite	Discharged home	
47	Control	34	First	Shortness of breath, general body pains, vomiting, weight loss, night sweats for 3 weeks	1	District	27	F	Y	N	8	63	136	Previously undiagnosed TB		Probable	Death	
48	Control	175	First	Fever and coughing for 2 days	68	District	39	M	Y	N	11.9	64	639	Previously undiagnosed TB	Bacterial infection	Probable	Discharged home	
49	Control	3	First	Headache, neck stiffness and vomiting.	15	District	31	M	N	N	12.3	32	67	AIDS related illness		Definite	Discharged home	
49	Control	87	Second	Headache and back pain and	4	District	32	M	Y	N	11.1	42	56	Surgical		Probable	Discharged home	

Pt No	TB Fast Track trial arm	Days from enrolment to admission	Admission order	Presenting problem	Duration of admission (days)	Hospital level	Age (yrs)	Sex	On ART	On TB tx	HB (g/dl)	CD4 (cells/ µL)	Creatinine µmol/L	Assigned cause of admission by primary physician	Assigned cause of admission by secondary physician	Likelihood of cause of admission	Outcome	Died within 90 days of discharge
				constipation														
49	Control	98	Third	Headache, painful neck and shoulders	28	District	32	M	Y	N	11.6	42	66	AIDS related illness		Definite	Discharged home	
50	Intervention	306	First	Diarrhoea and vomiting for 2 days	3	District	35	F	Y	N	8.3	22	152	AIDS related illness		Possible	Discharged home	
50	Intervention	328	Second	Diarrhoea and vomiting for 5 days	4	District	35	F	Y	N	6.6	22	80	AIDS related illness		Possible	Discharged home	
50	Intervention	342	Third	Diarrhoea and vomiting for 1 month	3	District	35	F	Y	N		22		AIDS related illness	AIDS-related illness	Possible	Discharged home	
50	Intervention	359	Fourth	Diarrhoea, fever and general body weakness	7	District	35	F	Y	N	6.8		68	AIDS related illness		Possible	Discharged home	
51	Intervention	7	First	Confusion, insomnia, coughing for 5 days	31	District	37	F	N	Y	10.4	3	76	AIDS related illness		Definite	Discharged home	Yes
51	Intervention	41	Second	Acute confusion and fever 3 days post discharge	3	District	37	F	N	Y	7		74	AIDS related illness		Probable	Death	
52	Intervention	112	First	Painful left eye with eye mass	2	District	43	F	Y	Y				Surgical		Probable	Discharged home	Yes
53	Intervention	12	First	Diarrhoea and vomiting	3	District	51	F	N	Y	8.6	78		On TB treatment with worsening TB disease	Gastrointestinal	Possible	Discharged home	
54	Intervention	12	First	Referred from clinic with positive serum cryptococcal antigen	6	District	49	F	N	N	12.9		63	AIDS related illness		Definite	Discharged home	
55	Intervention	39	First	Diarrhoea for 2 days	4	District	27	M	N	N	13.9	4	94	Gastrointestinal		Possible	Discharged home	
56	Intervention	25	First	Referred from clinic with positive serum cryptococcal antigen	3	District	27	F	N	N				AIDS related illness		Definite	Discharged home	
57	Intervention	8	First	Referred from	2	District	45	F	N	N	11.7	66	74	AIDS related illness		Definite	Discharged	

Pt No	TB Fast Track trial arm	Days from enrolment to admission	Admission order	Presenting problem	Duration of admission (days)	Hospital level	Age (yrs)	Sex	On ART	On TB tx	HB (g/dl)	CD4 (cells/ µL)	Creatinine µmol/L	Assigned cause of admission by primary physician	Assigned cause of admission by secondary physician	Likelihood of cause of admission	Outcome	Died within 90 days of discharge
				clinic with positive serum cryptococcal antigen													home	
58	Intervention	47	First	Drowsy for a week, loss of appetite, general weakness and vomiting	1	District	42	M	N	Y	10.4	1	770	On TB treatment with worsening TB disease		Definite	Death	
59	Intervention	7	First	Productive cough, loss of weight and appetite, night sweats for 3 weeks	2	District	23	F	N	Y	7.6	6	48	On TB treatment with worsening TB disease		Possible	Discharged home	
59	Intervention	37	Second	Chest pain, productive cough and headache	4	District	23	F	N	Y	8.6		32	Surgical		Definite	Discharged home	
59	Intervention	76	Third	Attempted suicide attempt	1	District	23	F	Y	N				Psychiatric		Probable	Discharged home	Yes
59	Intervention	134	Fourth	Loss of appetite and weight, diarrhoea, general body weakness and vomiting for 3 weeks	5	District	23	F	Y	Y	9.1		42	On TB treatment with worsening TB disease		Probable	Death	
60	Intervention	154	First	loss of appetite, diarrhoea, cough, general body weakness and painful legs	7	District	40	F	N	Y	8.8	30	154	AIDS related illness		Definite	Discharged home	
60	Intervention	204	Second	Diarrhoea and vomiting for 1 month	6	District	40	F	Y	Y	6.1		383	AIDS related illness		Definite	Discharged home	Yes
61	Intervention	4	First	Referred from clinic with positive serum cryptococcal antigen	1	District	44	M	N	Y	12.5	84	81	AIDS related illness		Definite	Discharged home	
62	Intervention	124	First	Diarrhoea and vomiting for 3 days	3	District	57	M	Y	Y	8.2		477	Gastrointestinal		Possible	Death	

Pt No	TB Fast Track trial arm	Days from enrolment to admission	Admission order	Presenting problem	Duration of admission (days)	Hospital level	Age (yrs)	Sex	On ART	On TB tx	HB (g/dl)	CD4 (cells/ µL)	Creatinine µmol/L	Assigned cause of admission by primary physician	Assigned cause of admission by secondary physician	Likelihood of cause of admission	Outcome	Died within 90 days of discharge
63	Intervention	14	First	Jaundice and vomiting for 3 days after starting TB treatment	3	District	32	F	N	Y	11.9	24	86	Treatment-related	Treatment-related	Probable	Discharged home	
64	Intervention	131	First	Genital warts	2	District	33	M	Y	N	17.4		76	Surgical		Definite	Discharged home	
65	Intervention	22	First	General body weakness and shortness of breath	7	District	45	F	N	Y	10.5		68	AIDS related illness	AIDS-related illness	Possible	Death	
66	Intervention	40	First	Jaundice, dark urine and general body weakness 3 weeks after starting TB treatment	4	District	31	F	N	Y	10.1	4	66	Treatment-related		Probable	Discharged home	
67	Intervention	16	First	General body weakness and unable to walk	5	District	56	M	N	N	7.4	34	153	Previously undiagnosed TB	Previously undiagnosed TB	Definite	Discharged home	
67	Intervention	41	Second	Jaundice and abdominal pain	2	District	56	M	N	Y	8.7		153	Treatment-related		Possible	Discharged home	
68	Intervention	146	First	Referred from clinic with positive serum cryptococcal antigen	10	District	48	M	N	Y	11.6	22	69	On TB treatment with worsening TB disease		Possible	Discharged home	
68	Intervention	224	Second	Abdominal pain and constipation	5	District	48	M	Y	Y	8.5		67	Surgical		Possible	Discharged home	
69	Intervention	188	First	Abdominal pain , dry cough and generalised weakness	15	District	47	M	Y	Y	12.7		88	On TB treatment with worsening TB disease		Probable	Discharged home	
70	Intervention	55	First	Shortness of breath for 2 days	22	District	25	F	Y	Y	10	36	90	On TB treatment with worsening TB disease		Definite	Discharged home	
71	Intervention	11	First	Referred from clinic with positive serum cryptococcal antigen	1	District	52	M	N	N	12.2	72	98	AIDS related illness		Definite	Discharged home	
72	Intervention	80	First	Cough and pleuritic chest	11	District	51	M	Y	Y	11.1		67	AIDS related illness		Definite	Discharged home	

Pt No	TB Fast Track trial arm	Days from enrolment to admission	Admission order	Presenting problem	Duration of admission (days)	Hospital level	Age (yrs)	Sex	On ART	On TB tx	HB (g/dl)	CD4 (cells/ µL)	Creatinine µmol/L	Assigned cause of admission by primary physician	Assigned cause of admission by secondary physician	Likelihood of cause of admission	Outcome	Died within 90 days of discharge
				pain														
72	Intervention	194	Second	Admitted for chemotherapy of lymphoma	10	District	51	M	Y	Y				AIDS related illness		Definite	Discharged home	
73	Intervention	15	First	Productive cough, loss of weight and appetite, night sweats for 4 weeks	13	District	62	M	N	Y	6.4		84	AIDS related illness		Definite	Discharged home	
74	Intervention	218	First	Diarrhoea, vomiting, and weakness for2 days	2	District	54	M	Y	N	2.5		393	Gastrointestinal		Probable	Discharged home	Yes
74	Intervention	236	Second	Diarrhoea and vomiting for 2 weeks	6	District	54	M	Y	N	6	54	62	AIDS related illness	AIDS-related illness	Possible	Discharged home	Yes
74	Intervention	273	Third	swollen painful leg	12	District	54	M	Y	N	8.6		77	Surgical		Definite	Death	
75	Intervention	8	First	Referred from clinic with positive serum cryptococcal antigen and elevated creatinine	6	District	41	M	N	N	12.3	8	258	AIDS related illness		Definite	Discharged home	
76	Intervention	275	First	Vaginal and anal lesions	14	District	30	F	Y	N	11.2	19		Surgical		Definite	Discharged home	Yes
77	Intervention	1	First	Lymphadenitis, dizziness, cough, loss of weight for 3 weeks	30	District	24	F	N	N	6.9	35	135	Previously undiagnosed TB	Previously undiagnosed TB	Probable	Discharged home	Yes
77	Intervention	90	Second	Painful feet, general body weakness and dyspnoea for 1 week	3	District	25	F	Y	Y	11.2		425	AIDS related illness		Definite	Death	
78	Intervention	74	First	Shortness of breath, general body weakness, chest pain	3	District	33	F	Y	Y	5.7			On TB treatment with worsening TB disease		Possible	Discharged home	Yes
79	Intervention	74	First	Epigastric pain,	9	District	44	M	Y	Y	5.3		147	Bacterial infection		Possible	Death	

Pt No	TB Fast Track trial arm	Days from enrolment to admission	Admission order	Presenting problem	Duration of admission (days)	Hospital level	Age (yrs)	Sex	On ART	On TB tx	HB (g/dl)	CD4 (cells/ µL)	Creatinine µmol/L	Assigned cause of admission by primary physician	Assigned cause of admission by secondary physician	Likelihood of cause of admission	Outcome	Died within 90 days of discharge
				painful legs, fevers, vomiting, jaundice														
80	Intervention	147	First	Difficulty in breathing	0	District	26	F	N	N	6.6		289	Bacterial infection		Possible	Death	
81	Intervention	13	First	Productive cough for 3 weeks	1	District	41	M	N	N	14.6	77	81	Non-communicable	Non-communicable	Possible	Discharged home	
82	Intervention	150	First	General weakness, fever and septic perineal wounds	9	District	33	F	Y	Y	4.9		87	Surgical	Bacterial infection	Definite	Discharged home	Yes
83	Intervention	73	First	Painful swollen legs and feet with purple skin lesions	17	District	23	F	Y	Y	6.6	324	45	AIDS related illness		Definite	Death	
84	Intervention	171	First	Elevated blood pressure in pregnancy	12	District	21	F	Y	N	11.2		56	Surgical	Surgical	Definite	Discharged home	
84	Intervention	189	Second	Elevated blood pressure in pregnancy	8	District	21	F	Y	N				Surgical		Definite	Discharged home	
85	Intervention	11	First	Referred from clinic with positive serum cryptococcal antigen	1	District	20	F	N	N		66	60	AIDS related illness		Definite	Discharged home	
86	Intervention	36	First	Jaundice, chest pain, painful swallowing for 5 days	14	District	46	F	Y	Y	11.6	314	187	Treatment-related	AIDS-related illness	Probable	Discharged home	
87	Control	30	First	Diarrhoea, cough, fatigue, weight loss for 2 weeks	2	District	33	F	Y	N	6.4		264	Previously undiagnosed TB		Possible	Death	
88	Control	61	First	Dyspnoea, coughing, diarrhoea and vomiting for 2 days	21	District	36	F	Y	N	6	50	876	Previously undiagnosed TB		Probable	Death	
89	Control	20	First	Painful lesion on finger for three months	5	District	44	F	Y	N	11.8		65	Surgical		Definite	Discharged home	
90	Control	295	First	General body weakness,	1	District	37	M	Y	N	6.9			Surgical		Probable	Death	

Pt No	TB Fast Track trial arm	Days from enrolment to admission	Admission order	Presenting problem	Duration of admission (days)	Hospital level	Age (yrs)	Sex	On ART	On TB tx	HB (g/dl)	CD4 (cells/ µL)	Creatinine µmol/L	Assigned cause of admission by primary physician	Assigned cause of admission by secondary physician	Likelihood of cause of admission	Outcome	Died within 90 days of discharge
				bedsores, shortness of breath for 3 days														
91	Control	221	First	Peripheral oedema and both legs painful for 1 week	8	District	32	M	Y	N	10.6		134	Treatment-related		Probable	Death	
92	Control	25	First	Shortness of breath, general body weakness, weight loss and diarrhoea	2	District	26	M	C	N	7		65	Bacterial infection		Probable	Death	
93	Control	22	First	Weakness, dehydration, difficulty in breathing	7	District	38	M	N	N	6.5		98	Previously undiagnosed TB		Probable	Discharged home	
94	Control	35	First	Diarrhoea for 2 days and vomiting for 3 weeks	5	District	57	M	Y	N	15	21	361	Gastrointestinal		Probable	Discharged home	
94	Control	249	Second	Fatigue, cough, difficulty breathing for 3 days	6	District	58	M	Y	N	9.6	6	655	End-organ failure		Probable	Death	
95	Control	337	First	Diarrhoea for 2 weeks, vomiting and general body weakness	3	District	65	F	Y	N	10.3	25	60	AIDS related illness		Definite	Discharged home	Yes
96	Control	158	First	Vomiting and diarrhoea for 3 days	10	District	32	F	Y	N	10.3			Gastrointestinal		Probable	Death	
97	Control	166	First	Cough, fever, chest pains, jaundice	4	District	43	M	Y	N	6.2		541	Bacterial infection		Probable	Death	
98	Control	41	First	Cough, weight and appetite loss, drenching night sweats for 1 week	4	District	30	M	N	N	6	100	105	Previously undiagnosed TB		Probable	Death	
99	Control	61	First	Decreased level of consciousness , vomiting for 3 days	3	District	44	M	Y	N	17.6		100	Non-communicable		Definite	Death	

Pt No	TB Fast Track trial arm	Days from enrolment to admission	Admission order	Presenting problem	Duration of admission (days)	Hospital level	Age (yrs)	Sex	On ART	On TB tx	HB (g/dl)	CD4 (cells/ µL)	Creatinine µmol/L	Assigned cause of admission by primary physician	Assigned cause of admission by secondary physician	Likelihood of cause of admission	Outcome	Died within 90 days of discharge
100	Control	322	First	Vomiting, epigastric pain and general weakness	9	District	34	F	Y	N	4.9	20	1028	End-organ failure		Probable	Death	
101	Control	1	First	Referred from clinic with positive serum cryptococcal antigen	6	District	35	M	Y	N	9	9	64	Previously undiagnosed TB		Possible	Discharged home	
102	Control	110	First	Vomiting and diarrhoea	4	District	29	F	Y	N	7.1			Previously undiagnosed TB	Gastrointestinal	Probable	Death	
103	Control	38	First	Loss of appetite and vomiting, confusion, loss of weight for 3 days	7	District	34	F	Y	Y	9.3		143	On TB treatment with worsening TB disease		Probable	Death	
104	Control	33	First	Vomiting and diarrhoea	8	District	39	M	Y	Y	6.7		688	On TB treatment with worsening TB disease	On TB treatment with worsening TB disease	Probable	Death	
105	Control	31	First	Coughing, night sweats, chest pain, vomiting and general body weakness for 2 weeks	7	District	23	F	Y	N	8			Previously undiagnosed TB	Previously undiagnosed TB	Possible	Death	
106	Intervention	106	First	Collapse with decreased level of consciousness	1	District	46	M	Y	Y				Bacterial infection		Possible	Death	
107	Intervention	41	First	Left leg swelling	10	District	51	M	Y	Y	11	52	105	Non-communicable		Definite	Discharged home	
107	Intervention	70	Second	Generalised tonic-clonic seizure	15	District	51	M	N	Y	12		74	Neurological		Probable	Discharged home	
107	Intervention	157	Third	Generalised tonic-clonic seizure	14	District	51	M	Y	Y			74	Neurological		Probable	Discharged home	
107	Intervention	232	Fourth	Generalised tonic-clonic seizure	10	District	52	M	Y	Y	15.2		124	Neurological	AIDS-related ilness	Probable	Discharged home	
107	Intervention	247	Fifth	Generalised tonic-clonic seizure	10	District	52	M	Y	Y	12.3			Neurological		Probable	Discharged home	
108	Intervention	120	First	Cough, generalised body weakness, haemoptysis and	8	District	51	F	Y	Y	11.16	59	65	On TB treatment with worsening TB disease		Possible	Discharged home	

Pt No	TB Fast Track trial arm	Days from enrolment to admission	Admission order	Presenting problem	Duration of admission (days)	Hospital level	Age (yrs)	Sex	On ART	On TB tx	HB (g/dl)	CD4 (cells/ µL)	Creatinine µmol/L	Assigned cause of admission by primary physician	Assigned cause of admission by secondary physician	Likelihood of cause of admission	Outcome	Died within 90 days of discharge
				night sweats for 2 weeks														
109	Intervention	83	First	Productive cough and chest pain	11	District	39	F	Y	Y	10.9	94	77	On TB treatment with worsening TB disease		Possible	Death	
110	Intervention	40	First	Generalised body weakness, dizziness, swollen legs, blurred vision	6	District	39	F	Y	N	10.6	29	66	Neurological		Possible	Discharged home	
111	Intervention	177	First	Headache, loss of weight and cough	10	District	56	M	Y	N	10.2	8	110	Previously undiagnosed TB		Definite	Discharged home	
112	Intervention	150	First	Confusion, diarrhoea, weak, chronically ill, jaundice for 2 days	9	District	48	F	Y	Y	11.3	33	142	On TB treatment with worsening TB disease	AIDS-related illness	Possible	Death	
113	Intervention	4	First	Difficulty in breathing, coughing for a week after 1 week of starting ART	3	District	27	F	Y	N	10.5			Previously undiagnosed TB	Previously undiagnosed TB	Definite	Discharged home	
113	Intervention	36	Second	Vomiting and diarrhoea	2	District	27	F	Y	N				Gastrointestinal		Possible	Discharged home	
113	Intervention	56	Third	Confused, neck stiffness and lymphadenopathy	25	District	27	F	Y	N	6.9	74	60	Previously undiagnosed TB		Possible	Discharged home	Yes
113	Intervention	127	Fourth	Confused and decreased level of consciousness	1	District	27	F	Y	Y	8.4		38	On TB treatment with worsening TB disease	Bacterial infection	Definite	Death	
114	Intervention	6	First	Diarrhoea and body weakness	10	District	65	M	N	Y		3	195	On TB treatment with worsening TB disease		Probable	Death	
115	Intervention	149	First	Unable to communicate, productive cough, body weakness for 4 days	11	District	64	F	Y	Y	7.9	32	250	Bacterial infection		Possible	Death	
116	Intervention	28	First	Dizziness, palpitations and fatigue	4	District	37	F	Y	Y	6.5	41		Haematological	Haematological	Definite	Discharged home	
116	Intervention	251	Second	Difficulty in breathing	14	District	38	F	Y	Y	7	30	58	On TB treatment with worsening TB disease		Possible	Discharged home	
117	Intervention	9	First	Admitted for chemotherapy of	5	District	42	F	C	Y	6.1			Non-communicable		Definite	Discharged home	Yes

Pt No	TB Fast Track trial arm	Days from enrolment to admission	Admission order	Presenting problem	Duration of admission (days)	Hospital level	Age (yrs)	Sex	On ART	On TB tx	HB (g/dl)	CD4 (cells/ μL)	Creatinine μmol/L	Assigned cause of admission by primary physician	Assigned cause of admission by secondary physician	Likelihood of cause of admission	Outcome	Died within 90 days of discharge
				breast cancer														
117	Intervention	85	Second	Deterioration of condition	0	District	42	F	C	Y				Non-communicable		Definite	Death	
118	Intervention	67	First	Decreased level of consciousness	9	District	32	F	Y	Y	13.8	14	83	On TB treatment with worsening TB disease		Definite	Death	
119	Intervention	7	First	Coughing, difficulty breathing, chronic abdominal pain	12	District	36	M	N	Y	8.8	61	179	On TB treatment with worsening TB disease		Possible	Discharged home	
120	Intervention	28	First	Visual loss for 2 weeks	30	District	25	F	N	Y	8.6	15	83	AIDS related illness		Probable	Death	
121	Intervention	26	First	Generalised body weakness, abdominal pain, jaundice, diarrhoea	1	District	25	M	Y	Y				Treatment-related		Possible	Death	
122	Intervention	31	First	Shortness of breath, dizziness and generalised weakness for 2 days	70	District	46	M	Y	N	3.9	7	72	Haematological		Definite	Discharged home	Yes
123	Intervention	34	First	Dyspnoea	5	District	34	F	Y	Y	6.3		72	AIDS related illness		Possible	Discharged home	
123	Intervention	174	Second	Recurrent perineal abscesses	5	District	35	F	Y	Y				Surgical		Probable	Discharged home	
124	Intervention	5	First	Diarrhoea, loss of appetite, loss of weight, cough for 1 week	2	District	39	M	N	Y	9.28		76	On TB treatment with worsening TB disease		Possible	Death	
125	Intervention	33	First	Shortness of breath, cough and vomiting for 2 weeks	7	District	35	M	Y	N	11.4	76	81	Previously undiagnosed TB		Definite	Discharged home	
126	Intervention	138	First	Severe abdominal pain, vomiting and constipation for 2 days	4	District	34	F	Y	N	9.8		54	Surgical		Possible	Discharged home	
127	Intervention	244	First	Pleuritic chest pain, cough, night sweats and loss of	4	District	34	F	N	N	6.7		139	Previously undiagnosed TB		Possible	Discharged home	

Pt No	TB Fast Track trial arm	Days from enrolment to admission	Admission order	Presenting problem	Duration of admission (days)	Hospital level	Age (yrs)	Sex	On ART	On TB tx	HB (g/dl)	CD4 (cells/ µL)	Creatinine µmol/L	Assigned cause of admission by primary physician	Assigned cause of admission by secondary physician	Likelihood of cause of admission	Outcome	Died within 90 days of discharge
				appetite														
128	Intervention	138	First	Abdominal and back pain for 1 month. Not eating for 5 days	18	District	50	M	N	Y	7.5		1606	End-organ failure	End-organ failure	Probable	Death	
129	Intervention	52	First	Dizziness for 2 days, chronic cough, night sweats, loss of weight, loss of appetite	17	District	38	F	Y	Y	2.4		237	Haematological		Probable	Discharged home	
130	Intervention	6	First	Dizziness, confused, loss of appetite	3	District	62	F	N	Y	9.5		814	Non-communicable		Possible	Death	
131	Intervention	12	First	Rash and fever for 1 week after starting TB 2 weeks before.	1	District	49	F	N	Y	13	26		Treatment-related		Probable	Discharged home	
132	Intervention	8	First	Difficulty breathing and coughing for 5 days	12	District	49	M	N	Y	9.4		320	AIDS related illness		Definite	Death	
133	Intervention	5	First	Seizure and headache	3	District	38	M	N	Y	13	80	53	Neurological		Probable	Discharged home	
134	Intervention	11	First	Urinary retention	5	District	56	M	N	N	12		88	Surgical		Definite	Discharged home	Yes
135	Intervention	151	First	Vomiting, weakness, ill with shortness of breath	9	District	53	F	Y	Y	7.3		103	Bacterial infection		Probable	Discharged home	
136	Intervention	85	First	Difficulty in breathing, generalised rash, painful eyes	1	District	35	F	Y	Y	2.7			Treatment-related		Probable	Death	
137	Intervention	12	First	General body pains, chest pains, loss of appetite, night sweats, productive cough	6	District	34	M	N	N	13.1		157	Previously undiagnosed TB		Probable	Discharged home	
138	Intervention	70	First	Cough, diarrhoea, vomiting, fever,	0	District	34	F	N	N				Previously undiagnosed TB		Probable	Death	

Pt No	TB Fast Track trial arm	Days from enrolment to admission	Admission order	Presenting problem	Duration of admission (days)	Hospital level	Age (yrs)	Sex	On ART	On TB tx	HB (g/dl)	CD4 (cells/ µL)	Creatinine µmol/L	Assigned cause of admission by primary physician	Assigned cause of admission by secondary physician	Likelihood of cause of admission	Outcome	Died within 90 days of discharge
				shortness of breath for 2 days														
139	Intervention	55	First	Diarrhoea and vomiting for 3 days, jaundice, itching for 2 weeks	1	District	57	F	Y	Y	9.8		1278	Treatment-related		Probable	Death	
140	Intervention	24	First	Referred from clinic with positive serum cryptococcal antigen	4	District	29	M	N	Y	11.3		114	AIDS related illness		Definite	Discharged home	
141	Intervention	10	First	Referred from clinic with positive serum cryptococcal antigen	5	District	38	M	N	Y	8.4	58	62	AIDS related illness		Definite	Discharged home	
142	Intervention	48	First	Difficulty in breathing, weakness, productive cough	22	District	52	M	Y	Y	5.2		1499	Bacterial infection		Definite	Death	
143	Intervention	21	First	Skin rash	15	District	36	M	N	Y	11.5		226	Treatment-related		Probable	Discharged home	
144	Intervention	20	First	Referred from clinic with positive serum cryptococcal antigen and headaches	14	District	61	F	N	N	11.3	78	108	AIDS related illness	AIDS related illness	Probable	Discharged home	
145	Intervention	361	First	Referred from clinic as patient was very pale	4	District	37	F	Y	N	4.1		56	Haematological		Definite	Discharged home	
146	Intervention	51	First	Body weakness, loss of appetite and weight	15	District	32	M	N	Y	4.5		394	End-organ failure	End-organ failure	Probable	Discharged home	
147	Intervention	0	First	Dyspnoea, dizziness, body weakness, painful feet for 4 weeks	11	District	35	M	N	N	4.3		102	Previously undiagnosed TB		Probable	Discharged home	Yes
147	Intervention	28	Second	Decreased level of consciousness and general weakness	5	District	35	M	N	Y	8.2		87	Treatment-related		Probable	Death	

Pt No	TB Fast Track trial arm	Days from enrolment to admission	Admission order	Presenting problem	Duration of admission (days)	Hospital level	Age (yrs)	Sex	On ART	On TB tx	HB (g/dl)	CD4 (cells/ µL)	Creatinine µmol/L	Assigned cause of admission by primary physician	Assigned cause of admission by secondary physician	Likelihood of cause of admission	Outcome	Died within 90 days of discharge
148	Intervention	54	First	General body weakness, loss of appetite, constipation, confusion	8	District	44	M	Y	N	9.2		517	Previously undiagnosed TB		Definite	Death	
149	Intervention	17	First	Referred from clinic with positive serum cryptococcal antigen and headaches	15	District	36	M	Y	Y	8	58	59	AIDS related illness		Definite	Discharged home	Yes
150	Intervention	77	First	Referred with high creatinine	7	District	42	M	N	Y	7.7	35	1018	End-organ failure		Possible	Discharged home	Yes
151	Intervention	41	First	Depressed level of consciousness and confusion	9	District	48	M	Y	N	8.4	9	290	Previously undiagnosed TB		Probable	Death	
152	Control	58	First	Coughing, vomiting, fever and unable to walk	4	District	55	M	Y	Y	12.4	271	65	On TB treatment with worsening TB disease		Possible	Discharged home	Yes
153	Control	31	First	Shortness of breath, fever with shivering and generalised body weakness. Coughing for 2 weeks	13	District	29	F	Y	N	10.1	78	109	Previously undiagnosed TB	Previously undiagnosed TB	Possible	Death	
154	Control	14	First	Weak and has diarrhoea	8	District	56	F	N	N	8.1	95	90	AIDS related illness	AIDS related illness	Possible	Discharged home	Yes
154	Control	37	Second	Diarrhoea	7	District	56	F	Y	N	7.8	97	166	AIDS related illness		Possible	Discharged home	Yes
155	Control	51	First	Mass on small palate (lymphoma)	26	District	42	F	Y	N	10.3		65	AIDS related illness	AIDS related illness	Definite	Discharged home	
155	Control	252	Second	Distention of abdomen and vomiting, shortness of breath	27	District	43	F	Y	N	8.5	15	104	Previously undiagnosed TB		Definite	Death	
156	Control	146	First	Generalised body weakness,	12	District	27	F	Y	N	5		59	Previously undiagnosed TB		Definite	Death	

Pt No	TB Fast Track trial arm	Days from enrolment to admission	Admission order	Presenting problem	Duration of admission (days)	Hospital level	Age (yrs)	Sex	On ART	On TB tx	HB (g/dl)	CD4 (cells/ µL)	Creatinine µmol/L	Assigned cause of admission by primary physician	Assigned cause of admission by secondary physician	Likelihood of cause of admission	Outcome	Died within 90 days of discharge
				dizziness, large lymph nodes														
157	Control	19	First	Hiccups for 3 days, generalised weakness and deterioration of condition	2	District	45	M	N	Y	11.5		74	On TB treatment with worsening TB disease		Possible	Discharged home	Yes
158	Control	30	First	Vomiting for 3 days, shortness of breath, fever	1	District	27	F	Y	N	9.9		65	Previously undiagnosed TB		Probable	Discharged home	
159	Control	6	First	Productive cough, difficulty breathing, loss of weight for 1 weeks	8	District	47	M	N	Y	10.7	6	50	On TB treatment with worsening TB disease	Bacterial infection	Definite	Death	
160	Control	132	First	Paralysis of left side of body for 3 days, slurred speech	18	District	36	M	Y	Y	10.9	39	58	Non-communicable		Probable	Death	
161	Control	34	First	Weakness, nausea and vomiting, jaundice for 1 week	4	District	31	F	Y	Y	7		133	Treatment-related		Probable	Discharged home	Yes
161	Control	73	Second	Leg pain and very pale	1	District	31	F	Y	Y	5.3		31	On TB treatment with worsening TB disease		Possible	Death	
162	Control	7	First	Generalised weakness, rigors, night sweats, cough, diarrhoea and loss of weight	3	District	27	M	N	N	11.7	36	136	Previously undiagnosed TB		Probable	Death	
163	Control	23	First	Vomiting and dizziness. One week on TB treatment	13	District	43	F	N	Y	11.9	47	120	Treatment-related		Probable	Discharged home	
163	Control	54	Second	Shortness of breath, coughing with fever, jaundice	8	District	43	F	Y	Y		21		On TB treatment with worsening TB disease		Possible	Discharged home	
163	Control	105	Third	Jaundice and dyspnoea	21	District	43	F	Y	Y				Treatment-related		Possible	Discharged home	
163	Control	228	Fourth	Admitted with	5	District	43	F	Y	Y				Surgical		Definite	Discharged	

Pt No	TB Fast Track trial arm	Days from enrolment to admission	Admission order	Presenting problem	Duration of admission (days)	Hospital level	Age (yrs)	Sex	On ART	On TB tx	HB (g/dl)	CD4 (cells/ µL)	Creatinine µmol/L	Assigned cause of admission by primary physician	Assigned cause of admission by secondary physician	Likelihood of cause of admission	Outcome	Died within 90 days of discharge
				cholelithiasis													home	
164	Control	40	First	Lower abdominal pain, dysuria, urinary frequency, back pain	6	District	36	F	Y	N	8.35		68	Bacterial infection		Probable	Discharged home	Yes
165	Control	79	First	Parasuicide with medicine overdose	3	District	28	M	Y	Y	13		60	Psychiatric	Psychiatric	Definite	Discharged home	
166	Control	57	First	General body weakness, dizziness, dyspnoea, fatigue, collapse	1	District	33	F	Y	N	3.7	3	58	Previously undiagnosed TB		Possible	Death	
167	Control	146	First	Diarrhoea, unable to communicate, weakness	13	District	37	F	Y	N	5.5		478	Previously undiagnosed TB		Possible	Death	
168	Control	17	First	Swollen left leg for 3 days	6	District	31	M	N	Y	9.3		69	Non-communicable		Definite	Discharged home	
169	Control	105	First	Collapse and weakness of left upper and lower limb	63	District	42	M	Y	N	12.2	62	66	Non-communicable		Definite	Death	
170	Control	14	First	3 week history of swollen right leg	9	District	47	F	N	N		75		Surgical		Possible	Discharged home	
171	Control	83	First	Referred with high creatinine	1	District	68	F	Y	N	12.3	73	291	End-organ failure	End-organ failure	Definite	Discharged home	
171	Control	327	Second	Confused and disorientated	3	District	69	F	Y	Y			577	End-organ failure		Probable	Discharged home	
172	Control	22	First	Confused for 1 week, diarrhoea for 3 days after 2 weeks of starting ART	1	District	36	M	Y	N	11.8		1788	Previously undiagnosed TB		Probable	Death	
173	Control	130	First	Diarrhoea and vomiting for 1 week	6	District	37	F	Y	Y	12.3	23	63	Gastrointestinal		Possible	Discharged home	
174	Control	127	First	Swollen right leg	1	District	33	M	Y	N				AIDS related illness		Definite	Discharged home	
174	Control	237	Second	Short of breath, fever, productive	9	District	34	M	Y	N	4.4	69	426	Previously undiagnosed TB		Possible	Discharged home	Yes

Pt No	TB Fast Track trial arm	Days from enrolment to admission	Admission order	Presenting problem	Duration of admission (days)	Hospital level	Age (yrs)	Sex	On ART	On TB tx	HB (g/dl)	CD4 (cells/ µL)	Creatinine µmol/L	Assigned cause of admission by primary physician	Assigned cause of admission by secondary physician	Likelihood of cause of admission	Outcome	Died within 90 days of discharge
				cough, chest pain, joint pains														
175	Control	59	First	Fever, jaundice, and abdominal pain	5	District	38	F	Y	N	8		94	Previously undiagnosed TB		Possible	Discharged home	
176	Control	232	First	Diarrhoea and vomiting	19	District	46	M	Y	N	6.2	10	1324	End-organ failure		Probable	Discharged home	
176	Control	330	Second	Presenting with shortness of breath, pedal oedema and fatigue	7	District	47	M	Y	N	2.7	71	434	End-organ failure		Probable	Discharged home	
177	Control	149	First	Poor appetite, fever, body weakness, weight loss, headache	12	District	48	M	Y	Y	12.9	48	67	On TB treatment with worsening TB disease		Probable	Death	
178	Control	244	First	Confusion	13	District	23	F	Y	Y	5.8	46	90	On TB treatment with worsening TB disease		Possible	Death	
179	Control	203	First	Shortness of breath, cough for 2 weeks, loss of weight and appetite and night sweats	1	District	37	M	Y	N				AIDS related illness		Definite	Discharged home	
180	Control	21	First	Confusion, headaches for 2 weeks and fever	19	District	39	M	Y	N			82	AIDS related illness	AIDS-related illness	Definite	Discharged home	
180	Control	202	Second	Headache, fever, weight loss and loss of appetite	5	District	40	M	Y	N	10.2		81	AIDS related illness	Previously undiagnosed TB	Probable	Discharged home	
181	Control	8	First	Unilateral painful swollen right limb for 3 weeks	6	District	39	F	N	N	10.1		116	Non-communicable	Non-communicable	Definite	Death	
182	Control	127	First	Generalised body weakness, confusion, fever	20	District	38	M	Y	Y	8.6	39	497	On TB treatment with worsening TB disease		Probable	Discharged home	Yes
182	Control	172	Second	Convulsions, headache, fever and general body weakness for 2 days	4	District	38	M	Y	Y	9.1		97	Surgical		Definite	Death	

Pt No	TB Fast Track trial arm	Days from enrolment to admission	Admission order	Presenting problem	Duration of admission (days)	Hospital level	Age (yrs)	Sex	On ART	On TB tx	HB (g/dl)	CD4 (cells/ µL)	Creatinine µmol/L	Assigned cause of admission by primary physician	Assigned cause of admission by secondary physician	Likelihood of cause of admission	Outcome	Died within 90 days of discharge
183	Control	39	First	Haemoptysis for 2 weeks	5	District	46	M	N	Y	6.8	52	92	Bacterial infection		Definite	Discharged home	
184	Control	61	First	Shortness of breath, confusion, fever, headache, cough and diarrhoea	2	District	40	M	Y	N	8.9		1826	End-organ failure		Probable	Death	
185	Control	168	First	Collapsed and difficulty in walking	7	District	33	M	Y	N	10.7		100	Treatment-related		Probable	Discharged home	Yes
185	Control	209	Second	Weakness and chronically ill	10	District	33	M	Y	N	4	74	335	Treatment-related		Probable	Discharged home	Yes
186	Control	18	First	Jaundice for 3 days and vomiting for 3 days	20	District	42	F	N	Y	9.6	13	82	Treatment-related		Probable	Discharged home	
187	Intervention	14	First	Vomiting for almost 2 weeks, fever, coughing	5	District	30	F	N	Y	11.7	4	64	On TB treatment with worsening TB disease		Possible	Discharged home	
188	Intervention	125	First	Painful right eye	2	District	32	F	Y	Y		124		Surgical		Probable	Discharged home	
189	Intervention	68	First	Loss of appetite, Loss of weight, vomiting, diarrhoea, weakness	34	District	40	F	Y	Y	4.8		113	Non-communicable	Non-communicable	Definite	Discharged home	
190	Intervention	15	First	Weight loss for 7 months, abdominal pain and distention for 6 days	1	District	42	F	N	N	6.1	110		Previously undiagnosed TB		Probable	Death	
191	Intervention	7	First	Productive cough, chest pain, shortness of breath for 2 weeks	4	District	46	M	Y	N	13.3	96	99	Previously undiagnosed TB		Probable	Discharged home	
192	Intervention	300	First	Hypoglycaemia	4	District	44	M	Y	Y	6	19	725	Bacterial infection		Definite	Death	
193	Intervention	8	First	Chest pain, dyspnoea, cough, tiredness and loss of appetite	41	District	54	M	N	Y	15.2	36	87	Non-communicable	Non-communicable	Definite	Discharged home	Yes
193	Intervention	69	Second	Cough with	16	District	54	M	Y	Y			49	Non-communicable		Definite	Death	

Pt No	TB Fast Track trial arm	Days from enrolment to admission	Admission order	Presenting problem	Duration of admission (days)	Hospital level	Age (yrs)	Sex	On ART	On TB tx	HB (g/dl)	CD4 (cells/ µL)	Creatinine µmol/L	Assigned cause of admission by primary physician	Assigned cause of admission by secondary physician	Likelihood of cause of admission	Outcome	Died within 90 days of discharge
				purulent sputum														
194	Intervention	36	First	Jaundice	14	District	41	M	Y	Y	9.9		76	On TB treatment with worsening TB disease	On TB treatment and worsening TB disease	Probable	Discharged home	
195	Intervention	14	First	Productive cough, loss of weight, night sweats, loss of appetite, vomiting	1	District	37	M	N	Y	10.7	81	94	On TB treatment with worsening TB disease		Possible	Discharged home	
196	Intervention	8	First	Difficulty breathing, coughing, diarrhoea	23	District	48	M	N	Y	9.1		68	Treatment-related		Probable	Discharged home	Yes
197	Intervention	42	First	Abdominal pain	7	District	43	F	N	Y	5.4		451	AIDS related illness		Definite	Discharged home	
198	Intervention	3	First	Shortness of breath, chest pain	4	District	30	M	N	Y	8.7	63	117	AIDS related illness		Possible	Discharged home	
199	Intervention	132	First	Swollen abdomen and pain for 2 weeks	8	District	27	F	Y	Y	7.5		535	On TB treatment with worsening TB disease		Possible	Discharged home	
199	Intervention	158	Second	Fatigue, dizziness, severe pallor	3	District	27	F	Y	Y	3.9		155	On TB treatment with worsening TB disease		Possible	Discharged home	
200	Intervention	204	First	Acute confusion, diarrhoea, poor appetite, loss of weight, fever and night sweats	11	District	52	M	Y	Y	4.3	13	106	Bacterial infection		Possible	Death	
201	Intervention	13	First	Chronic leg ulcer on the medial surface of leg	8	District	41	F	N	Y		66		Surgical	Surgical	Definite	Discharged home	
202	Intervention	22	First	Fever, generalised body weakness	4	District	52	M	N	Y	8.7	12		On TB treatment with worsening TB disease		Possible	Discharged home	
203	Intervention	15	First	Vomiting, loss of appetite, dizzy	2	District	35	M	N	Y	11.3	8	118	Gastrointestinal		Possible	Discharged home	
204	Intervention	113	First	Left sided weakness and slurred speech	1	District	42	M	Y	Y	12.7		49	AIDS related illness		Probable	Discharged home	Yes
205	Intervention	363	First	Septic hand for 1 week	4	District	29	F	Y	N	11.4	4	39	Surgical		Probable	Discharged home	Yes
206	Intervention	12	First	Unable to speak,	0	District	41	F	N	Y	8.8		1015	Surgical		Definite	Death	

Pt No	TB Fast Track trial arm	Days from enrolment to admission	Admission order	Presenting problem	Duration of admission (days)	Hospital level	Age (yrs)	Sex	On ART	On TB tx	HB (g/dl)	CD4 (cells/ µL)	Creatinine µmol/L	Assigned cause of admission by primary physician	Assigned cause of admission by secondary physician	Likelihood of cause of admission	Outcome	Died within 90 days of discharge
				vomiting for 1 day														
207	Intervention	44	First	Convulsions and decrease level of consciousness	17	District	53	M	N	Y	10	49	84	Neurological	AIDS related illness	Probable	Death	
208	Intervention	97	First	Vomiting and jaundice for 2 days	5	District	40	F	Y	Y	11.8		61	Treatment-related	Treatment-related	Possible	Discharged home	Yes
208	Intervention	126	Second	Vomiting, diarrhoea and general body weakness	1	District	40	F	Y	Y	14.5		73	Gastrointestinal		Possible	Discharged home	Yes
208	Intervention	142	Third	Weakness, vomiting, loss of weight for 4 days	20	District	40	F	Y	Y	10.6	9	62	On TB treatment with worsening TB disease		Probable	Death	
209	Intervention	223	First	Disorientated, difficulty in breathing, coughing, night sweats	3	District	49	M	N	Y	8.8	26	235	On TB treatment with worsening TB disease		Definite	Death	
210	Intervention	138	First	Severe abdominal pain	16	District	39	F	Y	Y	10.7		98	Surgical		Definite	Death	
211	Intervention	30	First	Vaginal bleeding for 3 months	2	District	35	F	Y	N	8.3	85	122	Surgical		Probable	Discharged home	
212	Intervention	4	First	Confused, neck pain, and fever	26	District	47	M	N	N	11.8	28	77	AIDS related illness		Definite	Discharged home	
213	Intervention	12	First	Referred from clinic with positive serum cryptococcal antigen and headaches	1	District	25	F	N	Y	9.1		65	AIDS related illness		Definite	Discharged home	
214	Control	25	First	Referred with swollen red eyes	1	District	60	F	C	N			84	Non-communicable		Definite	Discharged home	
215	Control	315	First	Diarrhoea and general body weakness	9	District	45	F	Y	N	10.4		371	AIDS related illness	AIDS related illness	Definite	Death	
216	Control	0	First	Pain on swallowing and vaginal bleeding	2	District	35	F	N	N		18		AIDS related illness		Possible	Discharged home	
217	Control	131	First	Diarrhoea and vomiting and	5	District	43	F	Y	N	4.1		2172	End-organ failure		Probable	Death	

Pt No	TB Fast Track trial arm	Days from enrolment to admission	Admission order	Presenting problem	Duration of admission (days)	Hospital level	Age (yrs)	Sex	On ART	On TB tx	HB (g/dl)	CD4 (cells/ µL)	Creatinine µmol/L	Assigned cause of admission by primary physician	Assigned cause of admission by secondary physician	Likelihood of cause of admission	Outcome	Died within 90 days of discharge
				generalised body weakness														
218	Control	129	First	Severe weakness, shortness of breath and rigors	14	District	34	M	Y	Y	7.4		180	On TB treatment with worsening TB disease		Probable	Discharged home	
218	Control	155	Second	Confusion	2	District	34	M	N	Y	9.4	26	83	On TB treatment with worsening TB disease		Probable	Discharged home	
219	Control	30	First	Pale and dizzy	4	District	28	M	Y	N	4	51	712	Haematological		Probable	Discharged home	
220	Control	26	First	Vomiting, lack of appetite, diarrhoea	4	District	37	F	Y	N	7.4		75	Previously undiagnosed TB		Probable	Death	
221	Control	11	First	Coughing, shortness of breath	4	District	40	M	N	Y	9		78	On TB treatment with worsening TB disease		Probable	Death	
222	Control	24	First	Weakness, confusion for 4 weeks	10	District	50	M	Y	N	15.1		67	AIDS related illness	AIDS related illness	Definite	Death	
223	Control	23	First	Vomiting, diarrhoea, dizziness for 1 week	1	District	34	F	Y	N				Gastrointestinal		Possible	Discharged home	
224	Control	19	First	Dizziness, vomiting and shortness of breath	1	District	36	F	Y	N	9.4		75	Bacterial infection	Bacterial infection	Possible	Discharged home	
225	Control	9	First	Headache, confusion, neck stiffness	23	District	32	M	N	N	13.2	14	90	Previously undiagnosed TB	AIDS related illness	Probable	Discharged home	
226	Control	7	First	Painful left leg	21	District	29	F	N	Y	9.7	64	58	Previously undiagnosed TB		Probable	Discharged home	
227	Intervention	250	First	Parasuicide with medicine overdose	2	District	26	F	Y	N	14.1		81	Psychiatric		Definite	Discharged home	
228	Intervention	125	First	Vomiting and diarrhoea	15	District	37	M	Y	Y	12.3		209	On TB treatment with worsening TB disease		Possible	Death	
229	Intervention	15	First	Vomiting, dysphagia, loss of appetite and weight	14	District	42	F	N	Y	9.2	6	263	AIDS related illness		Possible	Discharged home	Yes

Pt No	TB Fast Track trial arm	Days from enrolment to admission	Admission order	Presenting problem	Duration of admission (days)	Hospital level	Age (yrs)	Sex	On ART	On TB tx	HB (g/dl)	CD4 (cells/ µL)	Creatinine µmol/L	Assigned cause of admission by primary physician	Assigned cause of admission by secondary physician	Likelihood of cause of admission	Outcome	Died within 90 days of discharge
230	Intervention	7	First	Vomiting and general body weakness for 1 day	1	District	58	F	N	Y				On TB treatment with worsening TB disease		Possible	Death	
231	Intervention	39	First	Laceration and swelling of arm	2	District	52	M	Y	Y	9.7		252	Surgical		Possible	Death	
232	Intervention	283	First	Headache, tenderness of neck, diarrhoea and cough	1	District	33	M	Y	Y	10.3		81	AIDS related illness		Definite	Death	
233	Intervention	64	First	Diarrhoea, coughing, body weakness	9	District	39	F	Y	N	6.3	32	213	Previously undiagnosed TB	Previously undiagnosed TB	Probable	Death	
234	Intervention	7	First	Abdominal pain and distention, constipation and weakness for 4 days	1	District	31	F	N	Y	4.9		186	On TB treatment with worsening TB disease		Probable	Death	
235	Intervention	61	First	Parasuicide with medicine overdose	2	District	43	F	Y	Y				Psychiatric	Psychiatric	Probable	Discharged home	
236	Intervention	5	First	Confused, difficulty in breathing, weight loss and vomiting	3	District	46	M	N	Y	11		40	Unknown		Possible	Discharged home	Yes
237	Intervention	36	First	Cough, diarrhoea and fever	6	District	34	F	Y	Y				Unknown		Possible	Discharged home	
238	Intervention	120	First	Cough, difficulty in breathing, weight loss, fever and diarrhoea	13	District	35	F	Y	Y			40	Unknown		Possible	Discharged home	Yes
239	Intervention	2	First	Confusion, dyspnoea, vomiting, coughing	4	District	43	M	N	Y	8.2	78		Unknown		Possible	Discharged home	
240	Intervention	2	First	Confusion, skin rash, diarrhoea	4	District	45	M	N	Y				Unknown		Possible	Discharged home	
241	Control	7	First	Cough, weight loss, night sweats, vomiting, confused	2	District	43	M	Y	Y	7.2	58	256	On TB treatment with worsening TB disease		Possible	Death	

Pt No	TB Fast Track trial arm	Days from enrolment to admission	Admission order	Presenting problem	Duration of admission (days)	Hospital level	Age (yrs)	Sex	On ART	On TB tx	HB (g/dl)	CD4 (cells/ µL)	Creatinine µmol/L	Assigned cause of admission by primary physician	Assigned cause of admission by secondary physician	Likelihood of cause of admission	Outcome	Died within 90 days of discharge
242	Control	20	First	Lymphadenitis under arm	10	District	26	F	N	N	7.8		254	Previously undiagnosed TB	Previously undiagnosed TB	Possible	Discharged home	Yes
243	Control	244	First	Diarrhoea and vomiting, difficulty in breathing, weight loss for 2 weeks	1	District	42	M	Y	Y	7.8			Gastrointestinal	Gastrointestinal	Possible	Death	
244	Control	1	First	Genital sores, weakness, fever, coughing, urethral discharge	10	District	31	M	N	N	9.1		78	Unknown		Possible	Discharged home	
245	Control	85	First	Pregnant with abdominal pains	3	District	34	F	Y	N	10.2			Surgical		Definite	Discharged home	
246	Control	163	First	Dyspnoea, body weakness and pallor	2	District	42	F	Y	N	7.6		60	Haematological		Possible	Discharged home	
247	Control	2	First	Headache, pleuritic chest pain, night sweats	8	District	38	M	N	N	11.9		28	Previously undiagnosed TB		Possible	Death	
248	Control	19	First	Vomiting and diarrhoea for 1 week	16	District	51	F	Y	N	9.8	16	1557	End-organ failure		Probable	Death	
249	Control	17	First	Cough, swelling of right side of neck	19	District	43	F	Y	N	6.6		80	Previously undiagnosed TB		Definite	Discharged home	
250	Control	146	First	Tongue mass	5	District	43	F	Y	Y	11.6		54	Surgical		Probable	transferred to another facility	
251	Control	25	First	Shortness of breath and productive cough	2	District	40	F	N	N	5.4		1008	Bacterial infection		Probable	Discharged home	Yes

Supplementary table 3: Specific research-assigned causes of admission among those assigned an 'AIDS-related' cause, by probability (n = 55)

AIDS-related diagnosis	Total, n=55 (column %)	Probability of cause, n (row %)		
**Total**	55 (100.0)	33 (60.0)	7 (12.7)	15 (27.3)
Cryptococcaemia	11 (20.0)	11 (100)	0	0
Cryptococcal meningitis	11 (20.0)	7 (63.6)	4 (36.4)	0
Chronic gastroenteritis	11 (20.0)	2 (18.2)	0	9 (81.8)
Non-tuberculosis mycobacterial disease	7 (12.7)	5 (71.4)	0	2 (28.6)
Pneumocystis pneumonia	4 (7.3)	0	1 (25.0)	3 (75.0)
Kaposi Sarcoma	3 (5.5)	3 (100.0)	0	0
Lymphoma	3 (5.5)	3 (100.0)	0	0
Carcinoma of cervix	2 (3.6)	2 (100.0)	0	0
Cytomegalovirus retinitis	1 (1.8)	0	1 (100.0)	0
HIV encephalopathy	1 (1.8)	0	1 (100.0)	0
Oesophageal candidiasis	1 (1.8)	0	0	1 (100.0)

AIDS: Acquired Immune Deficiency Syndrome

Supplementary table 4: Research-assigned causes of subsequent admission (second, third, fourth, and fifth admissions), by probability (n = 53 admissions)

Admission cause category	Total, n (column %)	Probability of cause, n (row %)		
		Definite	Probable	Possible
**Total**	53 (100.0)	14 (26.4)	20 (37.7)	19 (35.9)
**AIDS-related illness**	14 (26.4)	4 (28.6)	3 (21.4)	7 (50.0)
**Previously undiagnosed TB**	3 (5.7)	1 (33.3)	0	2 (66.7)
**On TB treatment with worsening TB disease**	9 (17.0)	2 (22.2)	3 (33.3)	4 (44.4)
**Surgical**	8 (15.1)	5 (62.5)	2 (25.0)	1 (12.5)
**Treatment-related**	6 (11.3)	0	4(66.7)	2 (33.3)
**Non-communicable disease**	2 (3.8)	2 (100.0)	0	0
**Gastrointestinal disorders**	2 (3.8)	0	0	2 (100.0)
**Liver/renal failure**	3 (5.7)	0	3 (100.0)	0
**Neurological disorder**	5 (9.4)	0	4(80.0)	1(20.0)
**Psychiatric disorder**	1 (1.9)	0	1 (100.0)	0

AIDS: Acquired Immune Deficiency Syndrome; TB: tuberculosis

Supplementary table 5. Proportions of adults with advanced HIV who died as in-patients during first admission or within 90 days of discharge, by research-assigned cause of admission (n = 251 individuals)

Research-assigned cause of admission	Individuals admitted, n (column %)	Death in hospital or in the community within 90 days of first discharge, n (row %)	Death in hospital, n (row %)	Death within 90 days of discharge, n (row %)	Re-admitted within 30 days of discharge, n (row %)
**Any cause**	**251 (100)**	**137 (54.6)**	**98 (39.0)**	**39 (15.5)**	**19 (7.6)**
**Previously undiagnosed TB**	52 (20.7)	34 (65.4)	27 (51.9)	7 (13.5)	4 (7.7)
**On TB tx and worsening TB disease**	43 (17.1)	29 (67.4)	23 (53.5)	6 (14.0)	4 (9.3)
**AIDS-related illness**	41 (16.3)	15 (36.6)	8 (19.5)	7 (17.1)	4 (9.8)
**Bacterial infection**	20 (8.0)	16 (80.0)	13 (65.0)	3 (15.0)	0
**Surgical**	21 (8.4)	10 (47.6)	5 (23.8)	5 (23.8)	2 (9.5)
**Treatment-related**	18 (7.2)	9 (50.0)	5 (27.8)	4 (22.2)	2 (11.1)
**Non-communicable disease**	14 (5.6)	7 (50.0)	5 (35.7)	2 (14.3)	2 (14.3)
**Renal/liver failure**	9 (3.6)	6 (66.7)	5 (55.6)	1 (11.1)	0
**Gastrointestinal disorder**	12 (4.8)	5 (41.7)	4 (33.3)	1 (8.3)	1 (8.3)
**Unknown**	6 (2.4)	2 (33.3)	0	2 (33.3)	0
**Neurological disorder**	5 (2.0)	2 (40.0)	2 (40.0)	0	0
**Haematological disorder**	7 (2.8)	2 (28.6)	1 (14.3)	1 (14.3)	0
**Psychiatric disorder**	3 (1.2)	0	0	0	0

AIDS: acquired immune deficiency syndrome; TB: tuberculosis; tx: treatment

Supplementary table 6: Research-assigned causes of admission in adults admitted to hospital after enrolment to the TB Fast Track trial (Table 6A intervention group; Table 6B control group), shown by TB Fast Track algorithm-assessed probability for tuberculosis (based on TB symptoms*, hemoglobin level, LF-LAM result, and body mass index)

Table 6A: Intervention group (n = 145)

**Probability of TB based on TBFT algorithm†**	**High‡ (n = 91)**		**Medium§ (n = 35)**		**Low‖ (n = 19)**	
	**Yes, n (col %)**	**No, n (col %)**	**Yes, n (col %)**	**No, n (col %)**	**Yes, n (col %)**	**No, n (col %)**
**On TB treatment at admission**	74 (81.3)	17 (18.7)	23 (65.7)	12 (34.3)	4 (21.1)	15 (78.9)
Research-assigned cause of first admission	***n (%/74)***	***n (%/17)***	***n (%/23)***	***n (%/12)***	***n (%/4)***	***n (%/15)***
Previously Undiagnosed TB (16)	0	7 (41.2)	0	4 (33.3)	0	5 (33.3)
Worsening TB disease on TB treatment (30)	25 (33.8)	0	4 (17.4)	0	1 (25.0)	0
Bacterial infection (11)	6 (8.1)	1 (5.9)	3 (13.0)	0	0	1 (6.7)
AIDS-related illnesses (n=29)	15 (20.3)	3 (17.6)	5 (21.7)	6 (50.0)	0	0
Treatment-related conditions (12)	11 (14.9)	0	1 (4.3)	0	0	0
Non-communicable diseases (7)	3 (4.1)	0	3 (13.0)	1 (8.3)	0	0
Surgical conditions (14)	3 (4.1)	3 (17.6)	2 (8.7)	1 (8.3)	2 (50.0)	3 (20.0)
Psychiatric conditions (2)	1 (1.4)	1 (5.9)	0	0	0	0
Renal/liver failure (3)	2 (2.7)	0	1 (4.3)	0	0	0
Hematological (5)	2 (2.7)	1 (5.9)	0	0	0	2 (13.3)
Neurological (4)	1 (1.4)	0	1 (4.3)	0	0	2 (13.3)
Gastrointestinal (7)	1 (1.4)	1 (5.9)	2 (8.7)	0	1 (25.0)	2 (13.3)
Unknown (5)	4 (5.5)	0	1 (4.3)	0	0	0

* WHO TB symptoms: cough, fever, weight loss, and night sweats

† In the intervention group, TBFT probabilities were assessed in real-time and influenced management; in the control group, probabilities were assessed retrospectively and did not influence management

‡ TBFT High probability: LF-LAM positive or hemoglobin <10 g/dl or BMI <18.5 kg/m^2 ; TBFT management in trial group: TB treatment started as soon as possible

§ TBFT medium probability: ≥1 TB symptom, and hemoglobin >10 g/dl and BMI ≥18.5 kg/m^2; TBFT management in trial group: further investigation and review within 1 week.

‖ TBFT low probability: No TB symptoms, and LF-LAM negative and hemoglobin ≥10 g/dl and BMI ≥18.5 kg/m^2; TBFT management in the trial group: ART started as soon as possible.

¶ TBFT algorithm score unable to be allocated to individuals in the control group as missing defining

AIDS: Acquired Immune deficiency syndrome; ART: antiretroviral therapy; BMI: body mass index; LF-LAM: lateral-flow lipoarabinomannan; TB: tuberculosis; TBFT: TB Fast Track trial; WHO: World Health Organization

Table 6B: control group (n = 106)

Probability of TB based on TBFT algorithm†	High‡ (n = 53)		Medium§ (n = 16)		Low ‖ (n = 9)		Unknown ¶ (n=28)	
	Yes, n (col %)	No, n (col %)	Yes, n (col %)	No, n (col %)	Yes, n (col %)	No, n (col %)	Yes, n (col %)	No, n (col %)
**On TB treatment at admission**	13 (24.5)	40 (75.5)	4 (25.0)	12 (75.0)	1 (11.1)	8 (88.9)	7 (25.0)	21 (75.0)
Research-assigned cause of first admission	***n (%/13)***	***n (%/40)***	***n (%/4)***	***n (%/12)***	***n (%/1)***	***n (%/8)***	***N (%/7)***	***N (%/21)***
Previously Undiagnosed TB (36)	0	20 (50.0)	0	5 (41.7)	0	1 (12.5)	0	10 (47.6)
Worsening TB disease on TB treatment (13)	6 (46.2)	0	1 (25.0)	0	1 (100.0)	0	5 (71.4)	0
Bacterial infection (9)	1 (7.7)	4 (10.0)	0	2 (16.7)	0	1 (12.5)	0	1 (4.8)
AIDS-related illnesses (12)	0	4 (10.0)	0	1 (8.3)	0	4 (50.0)	0	3 (14.3)
Treatment-related conditions (6)	2 (15.4)	1 (2.5)	1 (25.0)	0	0	0	1 (14.3)	1 (4.8)
Non-communicable diseases (7)	2 (15.4)	1 (2.5)	1 (25.0)	1 (8.3)	0	1 (12.5)	0	1 (4.8)
Surgical conditions (7)	1 (7.7)	1 (2.5)	0	2 (16.7)	0	3 (37.5)	0	6 (28.6)
Psychiatric conditions (1)	0	0	0	0	0	0	1 14.3)	0
Renal/liver failure (6)	0	4 (10.0)	0	0	0	0	0	2 (9.5)
Hematological (2)	0	2 (5.0)	0	0	0	0	0	0
Neurological (1)	0	1 (2.5)	0	0	0	0	0	0
Gastrointestinal (5)	1 (7.7)	1 (2.5)	1 (25.0)	1 (8.3)	0	1 (12.5)	0	0
Unknown (1)	0	1 (2.5)	0	0	0	0	0	0

* WHO TB symptoms: cough, fever, weight loss, and night sweats
† In the intervention group, TBFT probabilities were assessed in real-time and influenced management; in the control group, probabilities were assessed retrospectively and did not influence management
‡ TBFT High probability: LF-LAM positive or hemoglobin <10 g/dl or BMI <18.5 kg/m^2 ; TBFT management in trial group: TB treatment started as soon as possible
§ TBFT medium probability: ≥1 TB symptom, and hemoglobin >10 g/dl and BMI ≥18.5 kg/m^2; TBFT management in trial group: further investigation and review within 1 week.
‖ TBFT low probability: No TB symptoms, and LF-LAM negative and hemoglobin ≥10 g/dl and BMI ≥18.5 kg/m^2; TBFT management in the trial group: ART started as soon as possible.
¶ TBFT algorithm score unable to be allocated to individuals in the control group as missing defining
AIDS: Acquired Immune deficiency syndrome; ART: antiretroviral therapy; BMI: body mass index; LF-LAM: lateral-flow lipoarabinomannan; TB: tuberculosis; TBFT: TB Fast Track trial; WHO: World Health Organization

Supplementary table 7: Selected characteristics of adults with advanced HIV who had sputum samples collected and chest radiographs performed during hospitalization in all admissions not on TB treatment (n = 144) compared to those with symptoms suggestive of TB and not on TB treatment (n = 57).

Characteristic	Admissions in people not on TB treatment (n = 144)			Admissions in people not on TB treatment who had symptoms suggestive of TB‡ (n = 57)		
	Overall, n, (column %)	Sputum sample test collected, n (row %)	Chest radiograph performed, n (row %)	Overall, n, (column %)	Sputum sample test collected, n (row %)	Chest radiograph performed, n (row %)
**All**	**144**	**18 (12.5)**	**59 (41.0)**	**57**	**12 (21.1)**	**37 (65.0)**
Age, years (median, IQR)	144	40.7 (34.5–43.7)	35.5 (32.5–41.8)	57	39.8 (30.7–43.3)	35.1 (32.3–41.4)
Female	80	8 (10.0)	29 (36.3)	26	5 (19.2)	17 (65.4)
Male	64	10 (15.6)	30 (46.9)	31	7 (22.6)	20 (64.5)
Province						
Gauteng	117	12 (10.3)	50 (42.7)	46	7 (15.2)	31 (67.4)
Limpopo	15	6 (40.0)	5 (33.3)	10	5 (50.0)	5 (50.0)
North-West	12	0	4 (33.3)	1	0	1 (100.0)
Level of hospital admitted to						
District	134	15 (11.2)	52 (38.8)	53	10 (18.9)	34 (64.2)
Tertiary	10	3 (30.0)	7 (70.0)	4	2 (50.0)	3 (75.0)
On ART*	103	11 (10.7)	41 (39.8)	38	8 (21.1)	24 (63.2)
Previous TB†	18	12 (66.7)	8 (44.4)	8	4 (50.0)	6 (75.0)
CD4 count at enrolment to TBFT (cells/µL)						
<50 cells/µL	76	14 (18.4)	35 (46.1)	33	9 (27.3)	21 (63.6)
50–100 cells/µL	40	1 (2.5)	13 (32.5)	13	0	9 (69.2)
100–150 cells/µL	28	3 (10.7)	11 (29.3)	11	3 (27.3)	7 (63.6)

*2 individuals did not have recorded ART status
†Previous TB prior to enrolment to the TB Fast Track trial.
‡Cough, fever, night sweats, or weight loss
ART: antiretroviral treatment; IQR: interquartile range; TB: tuberculosis; TBFT: TB Fast Track trial
